# One‐dimensional TiO_2_ Nanotube Photocatalysts for Solar Water Splitting

**DOI:** 10.1002/advs.201600152

**Published:** 2016-09-21

**Authors:** Mingzheng Ge, Qingsong Li, Chunyan Cao, Jianying Huang, Shuhui Li, Songnan Zhang, Zhong Chen, Keqin Zhang, Salem S. Al‐Deyab, Yuekun Lai

**Affiliations:** ^1^ National Engineering Laboratory for Modern SilkCollege of Textile and Clothing, EngineeringSoochow UniversitySuzhou215123P. R. China; ^2^ School of Materials Science and EngineeringNanyang Technological UniversitySingapore639798Singapore; ^3^ Petrochemical Research ChairDepartment of ChemistryCollege of ScienceKing Saud UniversityRiyadh11451Saudi Arabia

**Keywords:** modification, photocatalysis, photo/photoelectro‐catalytic water splitting, synthesis, TiO_2_ nanotubes

## Abstract

Hydrogen production from water splitting by photo/photoelectron‐catalytic process is a promising route to solve both fossil fuel depletion and environmental pollution at the same time. Titanium dioxide (TiO_2_) nanotubes have attracted much interest due to their large specific surface area and highly ordered structure, which has led to promising potential applications in photocatalytic degradation, photoreduction of CO_2_, water splitting, supercapacitors, dye‐sensitized solar cells, lithium‐ion batteries and biomedical devices. Nanotubes can be fabricated via facile hydrothermal method, solvothermal method, template technique and electrochemical anodic oxidation. In this report, we provide a comprehensive review on recent progress of the synthesis and modification of TiO_2_ nanotubes to be used for photo/photoelectro‐catalytic water splitting. The future development of TiO_2_ nanotubes is also discussed.

## 1 Introduction

The rapid development of globalization and industrialization has caused a series of serious environmental problems to the world's population.^1^, ^2^, ^3^, ^4^, ^5^ The supplies of fossil energy sources such as oil and gas are depleting exponentially and may be depleted within the next several decades if the current trend continues. The consumption of fossile fuels has also contaminated the environment, leading to global warming and climate changes due to the greenhouse effect.^6^, ^7^, ^8^, ^9^, ^10^ To address these concerns, there has been considerable attention devoted to the development of clean and renewable energy sources to meet the increasing energy demand.^11^, ^12^, ^13^, ^14^ Water splitting for hydrogen production in the presence of a semiconductor photocatalyst has been studied extensively as a potential method to provide hydrogen, a clean energy source. On exposure to sunlight, solar energy can be absorbed on photocatalysts to produce hydrogen and degrade pollutants at the same time, paving the way for producing clean energy source and creating a cleaner environment.^15^, ^16^, ^17^, ^18^, ^19^

Since the titanium dioxide (TiO_2_) was discovered for its potential for water photo­lysis by Fujishima and Honda in 1972,^20^ it has been widely investigated in photocatalytic degradation of pollutants,^21^, ^22^ photoreduction of CO_2_ into energy fuels,^23^, ^24^ water splitting,^25^, ^26^ supercapacitors,^27^, ^28^ dye‐sensitized/quantum dot‐sensitized/perovskite solar cells,^29^, ^30^, ^31^ lithium‐ion batteries,^32^, ^33^ biomedical devices^34^, ^35^ and self‐cleaning.^36^, ^37^ After Iijima et al. discovered carbon nanotubes (CNTs) in 1991,^38^ much more interests were activated in the one dimensional (1D) dimensional tubular nanomaterials. In contrast to CNTs, TiO_2_ nanotubes were readily fabricated by template‐assisted technique,^39^ solvothermal method,^40^, ^41^ hydrothermal method,^42^, ^43^ and electrochemical anodic oxidation.^44^, ^45^ Different fabrication methods have their own unique advantages and features. Since TiO_2_ nanotubes were first synthesized by Hoyer using template‐assisted method,^46^ the effects of fabrication factors, doping methods and applications have expanded rapidly.^47^, ^48^, ^49^, ^50^ However, there are still some intrinsic drawbacks that have limited the wide application of TiO_2_ nanotubes in some fields. On the one hand, wide band gap makes TiO_2_ (anatase: 3.2 eV, rutile: 3.0 eV) occupy only 3–5% of the total solar spectrum. On the other hand, fast recombination of photogenerated electron‐hole pairs also leads to decreased efficiency in the photo/photoelectro‐catalytic activity.^51^, ^52^, ^53^, ^54^, ^55^ Therefore, many works have been devoted to enlarging the photocatalytic active surface, forming Schottky junction or heterojunctions, and engineering the band structure to match particular energy levels. These efforts aim to widen the visible light absorption, separate the recombination of electron/holes for improved photo‐to‐energy conversion efficiency.^56^, ^57^, ^58^, ^59^, ^60^

**Scheme 1:**
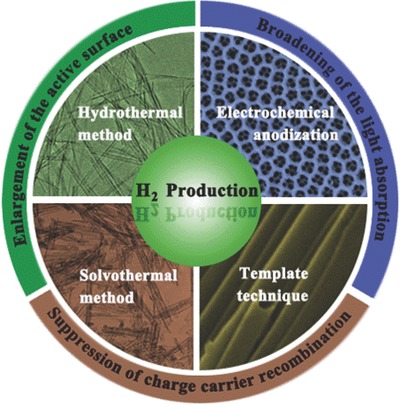
Schematic illustration of the preparation methods and modification strategies of TiO_2_ nanotubes for enhanced solar water splitting.

In this review, the preparation methods of TiO_2_ nanotube are summarized and the optimum conditions are proposed (**Scheme** **1**). Besides, this review will address various methods to modify TiO_2_ nanotube by enhancing the visible light absorption and suppressing the recombination of photogenerated electron/hole pairs. In addition, the mechanism and application of photo/photoelectro‐catalytic water splitting are comprehensively discussed. Challenges and the future development on 1D TiO_2_ nanotubes will be discussed in the end.

## 2 Basic Mechanism of Photo/photoelectro‐catalytic Water Splitting for Hydrogen Generation on TiO_2_ Nanotubes

**Figure 1:**
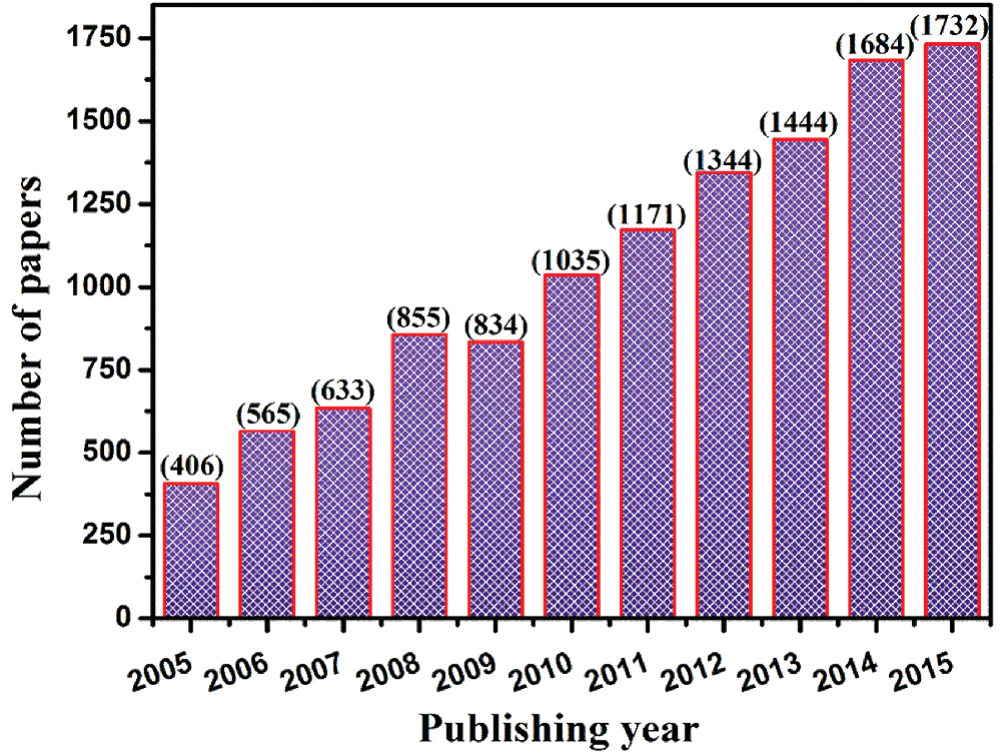
Number of articles published on photo/photoelectro‐catalytic water splitting for hydrogen production from 2005 to 2015 (Data was obtained from web of science database on March 31, 2016 using water splitting, hydrogen production and generation as key words).

Energy crisis and environmental issues are hot topics all over the word. Hydrogen is considered to be an ideal fuel for future energy demands because it can be produced in a sustainable manner and its consumption does not generate environmental pollutants. Besides, the photo/photoelectro‐catalytic water splitting reaction for hydrogen generation can be integrated with simultaneous pollutant removal in the oxidation process.^61^, ^62^, ^63^ After the early work of TiO_2_ photoelectrochemical hydrogen production reported by Fujishima and Honda, scientific and engineering interests in semiconductor photocatalysis have expanded tremendously.^64^, ^65^, ^66^, ^67^ The number of technical publications on water splitting for hydrogen production has grown rapidly; more than 10000 papers have been published in the last decade (**Figure** **1**).

**Figure 2:**
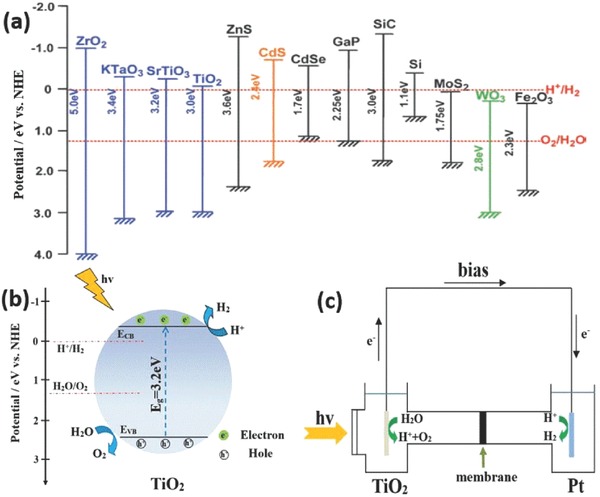
Relationship between band structure of semiconductor and redox potentials of water splitting (a). Schematic diagram for photocatalytic (b) and photoelectrocatalytic (c) water splitting on TiO_2_, respectively. (a) Reproduced with permission.^72^ Copyright 2009, American Chemical Society.

The mechanism of water splitting can be elaborated as follows. The water molecules are reduced by electrons to form H_2_, while they are also oxidized by holes to form O_2_ at the same time. The reduction potential (H^+^/H_2_) of water is 0 V and the oxidation potential (H_2_O/O_2_) is 1.23 V with respect to the normal hydrogen electrode (NHE). For hydrogen production, the conduction band (CB) level should be more negative than hydrogen production level (0 V) while the valence band (VB) should be more positive than water oxidation level (1.23 V) for efficient oxygen production from water by photo/photoelectro‐catalysis.^68^, ^69^, ^70^, ^71^ As we can see from band levels of various semiconductor materials (**Figure** **2**a), TiO_2_ is one of the most used semiconductors for hydrogen production as a result of their suitable band structures, low environmental impact and low toxicity, and high stability, while some other semiconductors such as SiC, CdS may not be suitable for water splitting because of the problem of photocorrsion.^72^, ^73^ In a photocatalytic water splitting process (Figure 2b), electrons at the valance band of TiO_2_ are exicted to the conduction band upon UV illumination. Hydrogen ions are reduced into hydrogen by the electrons at the conduction band, while the generated holes at the valence band oxide water molecules into oxygen (or degrade pollutants if they are presentin the solution). Apart from photocatalytic water splitting, photoelectrocatalytic water splitting has been proven a facile and promising route to solve the difficult problem of the fast recombination between photogenerated electron and holes, which can be beneficial for improving the photoelectrocatalytic hydrogen production efficiency. As depicted in Figure 2c, when a low bias potential is applied on the TiO_2_ nanotube, it can significantly facilitate the transfer of photocarriers and suppressed the recombination of photogenerated electrons and holes. Upon UV light irradiation, the electrons can leap up the valance band of TiO_2_ to the conduction band, and then be driven to the counter electrode via the external circuit, leaving the holes on the surface of the TiO_2_ nanotubes electrode. Meanwhile, a large number of active species were produced. The electrons at the counter electrode can react with hydrogen ions to generate hydrogen, while the holes at the valance band can react with water molecules to generate oxygen and hydrogen ions, or to degrade the pollutants, similar to the above discussed photocatlystic process.^74^, ^75^, ^76^, ^77^, ^78^, ^79^ The reaction mechanism is described as follows:

(1) $${\mathrm{TiO}}_{2}\;+\;\mathrm{hv}\;\to \;e^{-}\;+\;h^{+}$$

(2) $$2H^{+}\;+\;2e^{-}\;\to \;H_{2}$$

(3) $$2H_{2}O\;+\;4h^{+}\;\to \;O_{2}\;+\;4H^{+}$$

(4) $$\mathrm{Overall}\;\mathrm{reaction}:\;2H_{2}O\to O_{2}\;+\;2H_{2}$$

Compared to TiO_2_ nanoparticles, TiO_2_ nanotubes are widely used in water splitting due to their ordered tubular structure, strong ion‐exchange ability, relatively longer lifetime of electron/hole pairs.^80^, ^81^, ^82^, ^83^ However, the power conversion efficiency from solar to hydrogen by TiO_2_ nanotubes photo/photoelectro‐catalytic water splitting is still low, mainly due to fast (in absolute terms) recombination of electron/hole pairs and inability to utilize visible light. Therefore, continuous efforts have been made to solve these problems, which will be discussed in section 4.^84^, ^85^, ^86^

The photo/photoelectro‐catalytic activity for water splitting can be directly evaluated by the H_2_ or O_2_ gas evolution rate (μmol h^–1^ g^–1^ or μmol h^–1^ cm^–2^) and photocurrent density (mA cm^–2^). For photocatalytic systems, the time course and stoichiometry of H_2_ and O_2_ evolution should be taken into account for the evaluation of activity. In addition, for photoelectrocatalytic systems, the applied potential, wavelength and intensity of incident light should also be provided when performing an evaluation of activity. In order to compare the activity of photocatalysts under different reaction conditions, it is important to determine the overall quantum yield (QY) of TiO_2_ nanotube for water splitting.^87^ The overall QY is defined in Equation (5) and (6) for H_2_ and O_2_, respectively.^73^

(5) $$\mathrm{QY}\%\;=\;\frac{2\;\times \;\mathrm{Number}\;\mathrm{of}\;\mathrm{evolved}\;H_{2}\;\mathrm{molecules}}{\mathrm{Number}\;\mathrm{of}\;\mathrm{absorbed}\;\mathrm{photons}}\;\times \;100$$

(6) $$\mathrm{QY}\%\;=\;\frac{4\;\times \;\mathrm{Number}\;\mathrm{of}\;\mathrm{evolved}\;O_{2}\;\mathrm{molecules}}{\mathrm{Number}\;\mathrm{of}\;\mathrm{absorbed}\;\mathrm{photons}}\;\times \;100$$

Similarly, the incident photon to charge carrier generation efficiency (IPCE) in a photoelectrocatalytic system describes the ratio of effective photons or generated charges that generate electrochemical current to “incident photons” of monochromatic light. IPCE at different wavelengths is determined from the short circuit photocurrents (*I_sc_*) monitored at different excitation wavelengths (λ) to compare the photoresponse of the samples using Equation (7).^88^

(7) $$\mathrm{IPCE}\left(\%\right)\;=\;\frac{1240\;\times \;I_{sc}\left(\mathrm{mA}\;{\mathrm{cm}}^{-2}\right)}{\lambda \left(\mathrm{nm}\right)\;\times \;I_{inc}\left(\mathrm{mW}\;{\mathrm{cm}}^{-2}\right)}\;\times \;100$$

where *I_sc_* is the photocurrent density (mA cm^–2^) under illumination, λ is the wavelength (nm) of incident radiation, and *I_inc_* is the incident light power intensity on the TiO_2_ electrode (mW cm^–2^).

Besides, the turnover number (TON) for H_2_ generation is usually defined by the number of reacted molecules to that of an active site. However, it is often difficult to determine the number of active sites for photocatalysts. Therefore, the number of reacted electrons to the number of atoms in TiO_2_ nanotube (Equation (8)) or on the surface of TiO_2_ nanotube (Equation (9)) is employed as the TON.^72^

(8) $$\mathrm{TON}\;=\;\frac{\mathrm{Number}\;\mathrm{of}\;\mathrm{reacted}\;\mathrm{electrons}}{\mathrm{Number}\;\mathrm{of}\;\mathrm{atoms}\;\mathrm{in}\;a\;\mathrm{photocatalyst}}$$

(9) $$\mathrm{TON}\;=\;\frac{\mathrm{Number}\;\mathrm{of}\;\mathrm{reacted}\;\mathrm{electrons}}{\mathrm{Number}\;\mathrm{of}\;\mathrm{surface}\;\mathrm{atoms}\;\mathrm{in}\;a\;\mathrm{photocatalyst}}$$

It should be noteworthy that the quantum yield and turnover number is different from the solar energy conversion efficiency that is usually used for evaluation of hydrogen production activity. The overall conversion of solar energy is given by the following equation:^72^

(10) $$\mathrm{Solar}\;\mathrm{energy}\;\mathrm{conversion}(\%)\;=\;\frac{\mathrm{Output}\;\mathrm{energy}\;\mathrm{as}\;H_{2}}{\mathrm{Energy}\;\mathrm{of}\;\mathrm{incident}\;\mathrm{solar}\;\mathrm{light}}\;$$

## 3 Processing Techniques

Microstructure of TiO_2_ nanotubes plays a key role in their properties and photocatalytic efficiency. Various methods have been developed to prepare 1D TiO_2_ nanotubes in the past. In this section, we briefly introduce several main preparation methods, namely, the hydrothermal, solvothermal, electrochemical anodization, and template‐assisted method. Each fabrication method has unique advantages and functional features, comparison among these approaches is summarized in **Table** **1**.^89^

**Table 1:** Comparison of available methods for TiO_2_ nanotubes preparation

Fabrication method	Reaction conditions	Advantages	Disadvantages
Hydrothermal method	High pressure and high temperature.	High nanotube production rate.	Long reaction duration.
	Aqueous based solvent.	Easy to enhance the features of titanium nanotubes.	Difficult to achieve uniform size.
Solvothermal method	High pressure and high temperature.	Better control of the nanosize, crystal phase and narrow size distribution. Varieties of chosen organic solvent.	Critical reaction conditions.
	Organic solvent.		Long reaction time.
Electrochemical anodization method	5–50 V and 0.2–10 h under ambient conditions.	Ordered alignment with high aspect ratio.	Limited mass production.
	F^–^‐based buffered electrolytes and organic electrolytes, F^–^‐free electrolytes.	Controllable dimension of nanotubes by varying the voltage, electrolyte, pH and anodizing time.	Length distribution and separation of nanotubes over a large surface area is not well‐developed.
Template method	AAO, ZnO etc. as sacrificial template under specific conditions.	Controllable scale of nanotube by applied template.	Complicated fabrication process. Contamination or destroy of tubes may occur during fabrication process.
		Uniform size of nanotubes.	

### 3.1 Hydrothermal Method

Hydrothermal is an advanced nanostructural material processing technique encompassing the crystal growth, crystal transformation, phase equilibrium, and final ultrafine crystals formation.^90^ The hydrothermal method is the most widely used method for fabrication of 1D TiO_2_ nanostructures due to its simplicity and high productivity. Since the fabrication of TiO_2_‐based nanotubular materials through hydrothermal method by treating amorphous TiO_2_ powder at high temperatures in a highly concentrated NaOH solution without sacrificial templates was reported by Kasuga et al. for the first time in 1998,^91^ many efforts have been made on the synthesis of TiO_2_ nanotubes in such way.^92^, ^93^

**Figure 3:**
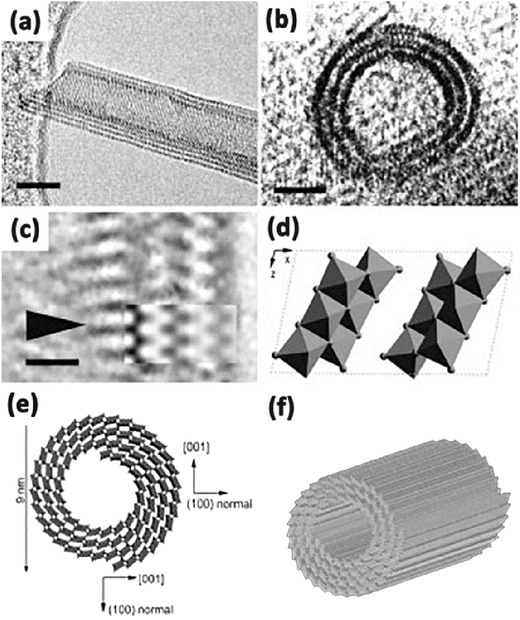
HRTEM image (a,b) of the multilayers TiO_2_ nanotubes. Enlarged HRTEM image (c) of a part in (a). Structure model of one‐unit cell of H_2_Ti_3_O_7_ on the [010] projection (d). Schematic drawing of the structure of nanotubes (e,f). Reproduced with permission.^94^

In a typical synthesis process, precursors of TiO_2_ and reaction solutions are mixed and enclosed in a stainless steel vessel under controlled temperature and pressure. After the reaction is complete, rinse with deionized water and acidic solution is needed to remove the impurities. Usually, there is a nearly 100% conversion for the precursors to TiO_2_ nanotubes in one single hydrothermal process. The morphologies of the obtained TiO_2_ depend on the process parameters, such as the structure of raw materials, the concentration of reacting solutions, reaction temperature and time, and even the acid washing. That is to say, the synthesis is controllable, hollow structure of the nanotubes with several layers via a single alkali treatment can be achieved (**Figure** **3**).^94^

The synthesis method can be classified into the acid‐hydrothermal and alkali‐hydrothermal approaches according to the reactants used for the hydrothermal synthesis of 1D TiO_2_ nanostructures. In the former method, the reactants are usually titanium salts with inorganic acid, and normally this method leads to the formation of TiO_2_ nanorods. Accordingly, the reactants in the latter method are generally TiO_2_ nanoparticles reacting in sodium hydroxide solution. The dissolution‐recrystallization is always involved in this process and the products include nanotubes or nanowires. These two methods have different reaction mechanisms, which produce different morphology and crystalline phases of the product in the 1D nanostructures.

**Figure 4:**
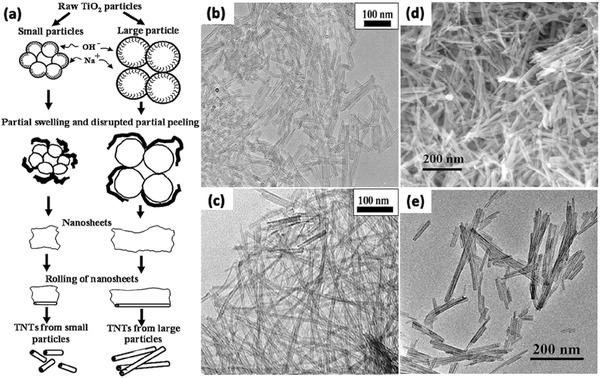
Schematic model of formation of TNTs from different size of raw TiO_2_ particles (a). TEM images of TiO_2_ nanotubes synthesized at reaction temperature of 120 °C with sonication power of 0 W (b) and 7.6 W (c). FE‐SEM (d) and TEM (e) images of the rutile/TiO_2_ nanotubes. (a–c) Reproduced with permission.^95^ Copyright 2009, Elsevier. (d,e) Reproduced with permission.^98^ Copyright 2013, Elsevier.

**Figure 5:**
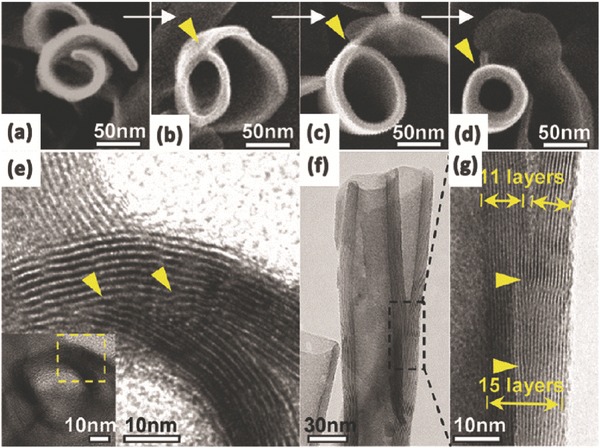
SEM images of the morphological evolution from a nanosheet into a nanotube (a–d); TEM image of a joint of a scrolled nanosheet in cross‐section (e); side‐view TEM image of a nanotube with an undetached exfoliating nanosheet (f) and its magnified TEM image (g). Reproduced with permission.^99^ Copyright 2011, American Chemical Society.

Though the hydrothermal process is a cost‐effective method, and the as‐prepared nanotubes have good dispersibility and high purity showing great potential for the formation of TiO_2_ nanostructures, some shortcomings still should not be ignored which limit its applications. For example, slow reaction kinetics result in long reaction time and limited length of the nanotubes. And nanotubes prepared are always non‐uniform on a large‐scale. For improvement, various approaches have been explored, such as ultrasonication assisted, microwave‐assisted, and rotation‐assisted hydrothermal methods. Nawin et al. has proved that the length of TiO_2_ nanotube becomes longer by the hydrothermal process coupled with sonication pretreatment.^95^ By varying size of raw TiO_2_ powder (400 nm and 1 μm), reaction temperature and sonication power, morphology of TiO_2_ nanostructures, length of TiO_2_ nanotube could be easily adjusted (**Figure** **4**a–c). TiO_2_ with various morphologies such as whiskers, nanotubes and nanofibers can be obtained at different sonication powers.^96^, ^97^ Huang et al. demonstrated a new method based on microwave irradiation for the preparation of rutile/titania‐nanotube composites that exhibit highly efficiency in visible light induced photocatalysis (Figure 4d,e).^98^ The nanocomposites exhibit multilayer‐wall morphologies with open‐ended cylindrical structures, and the presence of the rutile phase in the TiO_2_ nanotubes enhanced the light‐harvesting efficiency in photocatalytic reactions. In 2011, a facile vapor phase hydrothermal method for direct growth of vertically aligned TiO_2_ nanotubes with larger diameter on a titanium foil substrate was reported for the first time by Zhao et al.^99^ The resultant nanotubes consisted of more than 10 titanate layers, and displayed external diameters of 50–80 nm with an average wall thickness of 10 nm. Typically, unlike the mechanism frequently encountered in conventional liquid‐phase hydrothermal reactions, a distinctive nanosheet roll up mechanism can be observed during the nanotubes formation process (**Figure** **5**a–g). Torrente et al. synthesized TiO_2_ nanotubes with even longer length than conventional method at a low revolving speed.^100^ In particular, Tang et al. reported a new process to grew elongated TiO_2_ nanotubes with length up to tens of micrometers by a stirring hydrothermal method.^101^ This study confirmed that the mechanical force‐driven stirring process simultaneously improved the diffusion and surface reaction rate of TiO_2_ nanocrystal growth in solution phase, and the nanotube aspect ratio is strongly related to the precursor and the stirring rate. This method has provided 1D TiO_2_‐based nanotubular materials for long‐life and ultrafast rechargeable lithium‐ion batteries.

### 3.2 Solvothermal Method

**Figure 6:**
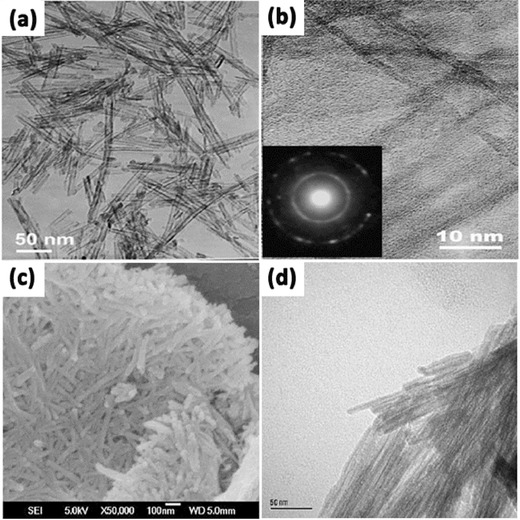
TEM images (a,b) at different magnifications of TiO_2_ synthesized by the solvothermal method (inset of b: electron diffraction pattern of a single TiO_2_ nanotube). SEM (c) and TEM (d) image of N‐doped TiO_2_ nanotubes fabricated by the solvothermal method. (a,b) Reproduced with permission.^110^ Copyright 2006, American Chemical Society. (c,d) Reproduced with permission.^111^ Copyright 2008, Royal Society of Chemistry.

The solvothermal method is also widely used in synthesizing metallic oxides such as ZrO_2_, ZnO, and Fe_3_O_4_ etc., and it is almost identical to the hydrothermal method except that the aqueous solutions used here are organic solvents.^102^, ^103^, ^104^ However, the temperature and pressure can be controlled at a higher level than that in hydrothermal method because a variety of chosen organic solvent have higher boiling points, thus it has better control with respect to the nanosize, crystal phase, size distribution and agglomeration of products.^105^, ^106^ The solvent plays a key role in determining the crystal morphology. Solvents with different physical and chemical properties can influence the solubility, reactivity, the diffusion behavior of the reactants, and the crystallization behavior of the final products. For example, TiO_2_ nanorods could be prepared by hydrothermal treatment of a titanium trichloride aqueous solution with NaCl at 160 °C for 2 h,^107^ while when TiO_2_ powders are put into NaOH aqueous solution and held at 20–110 °C for 20 h in an autoclave, TiO_2_ nanotubes are obtained.^91^ Typically, the solvothermal synthesis is usually conducted in an organic solvent such as ethanol and ethylene glycol, while the hydrothermal method reacts in water solutions.^108^, ^109^ Wang et al. successfully synthesized open‐ended TiO_2_ nanotubes by the solvothermal method using glycerol as solvents (**Figure** **6**a,b).^110^ The nanotubes consisted of continuous bilayers or multilayers (Figure 6b); this indicated that the nanotubes may probably be formed by scrolling conjoined multilayer nanosheets. And it exhibited a favorable discharge performance as anode materials in the application of lithium‐ion batteries. Zheng et al. prepared visible‐light‐responsive N‐doped TiO_2_ nanotubes via an environment‐conscious solvothermal treatment of protonated titanate nanotubes in an NH_4_Cl/ethanol/water solution at modest temperatures (Figure 6c,d).^111^ The obtained N‐doped TiO_2_ nanotubes are thermally stable and robust for photodegradation of methylene blue under visible light irradiation.

### 3.3 Electrochemical Anodization Method

The anodization method can be effectively employed to fabricate in situ nanotube arrays and has been widely used in anodizing various metals, such as 1D TiO_2_ nanotube arrays (TiO_2_ NTAs). Compared with the nanotubes constructed by the hydrothermal method, the size controllable TiO_2_ nanotube arrays fabricated by the anodization method are highly ordered and oriented perpendicular to the surface of the electrode substrate. Typically, these TiO_2_ NTAs are ideal photoanode materials which have been used for a long time due to its excellent stability, non‐toxicity, recyclability and cost‐effectivity.

In a typical experiment, a clean Ti plate is anodized in a fluoride‐based electrolyte (HF, NaF, or KF) using platinum as counter electrode under 10–25 V for 10–30 min.^112^ Usually, acetic acid, HNO_3_, H_3_PO_4_, H_2_SO_4_ or citric acid are used to adjust the acidity.^106^ Crystallized TiO_2_ nanotubes are obtained after the anodized Ti plate is annealed at 300–500 °C for 1–6 h in oxygen.^113^ In general, the length and wall thickness of the TiO_2_ NTAs could be controlled over a wide range with the applied potential, electrolyte type and concentration, pH, and temperature etc.^114^ The first known report on porous anodized TiO_2_ was published by Assefpour‐Dezfuly et al. in 1984, where the Ti metal was etched in alkaline peroxide at first, and then anodized in chromic acid.^115^ After that, Zwilling et al. reported the formation of self‐organized porous/tubular TiO_2_ structures in fluoride‐based electrolyte in 1999,^116^, ^117^ greatly prompting the development of tubular TiO_2_ structures.

The length of nanotubes in the first generation is limited to only approximately 500 nm or less, and it could not meet the requirement for some specific applications. In 2001, Grimes et al. first reported the preparation of self‐organized TiO_2_ nanotube arrays by utilizing anodization of titanium foil in a H_2_O‐HF electrolyte at room temperature.^118^ Subsequently, buffered neutral electrolytes containing various fluoride salts such as NaF or NH_4_F had been developed to prepare the second generation of nanotubes which have longer lengths up to several micrometers.^119^, ^120^, ^121^, ^122^, ^123^ It is noted that the nanotube length was increased to about 7 μm by controlling the pH of the anodization electrolyte and reducing the chemical dissolution of TiO_2_ during anodization.^124^ Later, the third generation TiO_2_ NTAs were anodized in non‐aqueous organic solvent electrolyte containing F^–^ with smooth and taller nanotube morphologies. Various organic solvents such as glycerol, ethylene glycol, formamide, and dimethyl sulfoxide (DMSO) had been used for the formation of TiO_2_ nanotubes with lengths up to approximately hundreds of micrometers.^125^, ^126^, ^127^, ^128^, ^129^ Some efforts have been made to produce TiO_2_ NTAs in a fluorine‐free electrolyte such as HClO_4_‐containing electrolyte, and they are commonly considered as the fourth‐generation nanotubes.^130^, ^131^ In addition, hexagonal nanotubes close packed arrays with high order via a multi‐step approach were achieved by Schmuki and Shin et al.,^132^, ^133^ and it was also thought as the fifth generation nanotubes. Since then, much effort has been made to optimize experimental parameters with different electrolytes or reacting conditions in order to efficiently achieve high quality self‐organized TNAs.^134^, ^135^, ^136^

**Figure 7:**
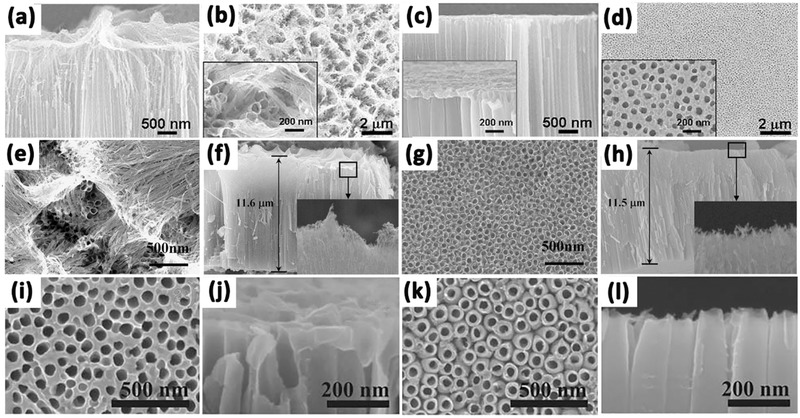
Side‐view (a) and top‐view (b) SEM images of TiO_2_ nanotube layers anodized in ethylene glycol and HF on non‐polished Ti plate. Side‐view (c) and top‐view (d) images of TiO_2_ nanotube layers anodized in ethylene glycol and HF on polished Ti plate. SEM images of TiO_2_ nanotube layers anodized in ethylene glycol and NH_4_F without (e,f) and with (g,h) protective layer. Top view (i) and side view (j) of the sample anodized under conventional method; top view (k) and side view (l) of the sample anodized using the new method at 80 °C post‐baking temperature for 10 min. (a–d) Reproduced with permission.^142^ Copyright 2008, Elsevier. (e–h) Reproduced with permission.^143^ Copyright 2009, The Electrochemical Society. (i–l) Reproduced with permission.^144^

In general, the anodization time and etching rate decide the tube length and nanotube‐layer thickness, while the dia­meter of the nanotubes is controlled linearly by the applied voltage.^137^, ^138^, ^139^, ^140^ And the nanotubes grown from organic electrolytes, such as ethylene glycol, glycerol, or ionic liquids, show some significant differences in morphology and composition compared with nanotubes grown in F^–^‐based aqueous electrolytes.^141^ For example, tube length is limited to 500–600 nm in electrolytes at low pH. In neutral electrolyte systems, the reduced chemical dissolution can lead to layer thicknesses of up to 2–4 μm. While in some glycerol or ethylene glycol based systems, reduced water content can further decrease chemical dissolution, thus the growth of tube could be significantly extended.^137^ Importantly, etching of the tube at their top could form grass‐like morphologies, which lead to inhomogeneous top structures (**Figure** **7**a,b,e,f,i,j). The collapsed and bundled tube tops mainly because the walls become too thin to support the capillary forces or their own weight during drying. The morphology of either thinned or partially dissolved tube walls is not favorable for carrier transport, and limits the energy conversion efficiency, thus efforts must be put forward to avoid this irregular nanotube structure during anodization. Kim et al. demonstrated a simple approach to prevent this effect by pretreating the polished Ti surfaces in F‐containing electrolytes to form a comparably compact rutile layer in the initial stages of anodization.^142^ This layer shows a comparably high resistance to chemical etching and can efficiently protect the tube tops, and thus allow the growth of nanotube layers with highly ordered and defined morphology in the subsequent tube growth process (Figure 7c,d). This ordered “nanograss”‐free tubes show significantly increased photocurrents and conversion efficiencies in dye‐sensitized solar cells. Similarly, Song et al. obtained anodic self‐organized TiO_2_ nanotube layers with a significantly improved tube morphology by forming a rutile layer on the Ti substrate by high temperature oxidation in air before the anodic tube growth.^143^ As a result, high aspect ratio nanotubes can be grown with an intact tube top (Figure 7g,h). In addition, Albu's group reported a very simple approach to produce anodic TiO_2_ nanotube arrays with highly defined and ordered tube openings by coating the surface with a photoresist that is slowly soluble in the electrolyte.^144^ With the thin layer of photoresist acting as a sacrificial initiation layer, open and “grass”‐free TiO_2_ nanotubes with clear tubular shape could be easily synthesized (Figure 7k,l).

**Figure 8:**
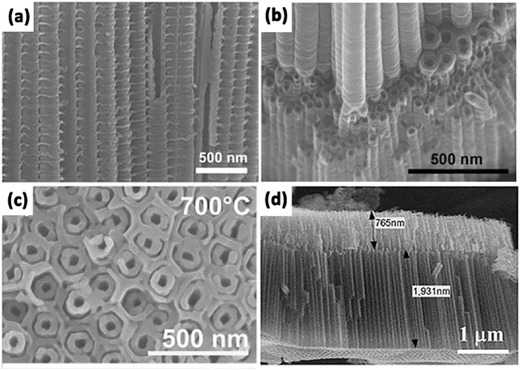
(a) Bamboo nanotubes fabricated by alternating voltage anodization. (b) branched nanotubes by voltage stepping. (c) double‐walled nanotubes. (d) amphiphilic double‐layer tubes.(a,b) Reproduced with permission.^145^ (c) Reproduced with permission.^148^ (d) Reproduced with permission.^149^ Copyright 2009, American Chemical Society.

Based on the chemical etching mechanism, by changing the anodization conditions, such as the applied voltage during the tube growth process, various modifications in the tube geometry can be achieved. For example, bamboo‐like stratification layers can be generated when stepping first to a lower voltage for a time and then stepping back to the original high voltage (**Figure** **8**a).^145^, ^146^ If the voltage is lowered during anodization, tube growth will be stopped or drastically slowed down. And at some point the oxide is thinned down sufficiently to continue growth under lower field conditions (the tube dia­meter will in this case adjust to the lower field and thus tube branching may occur (Figure 8b) owing to the permanent etching of the tube bottom in the fluoride environment. If tube wall separation (tube splitting) is faster than the etching process through the tube bottom, then second tube layer will initiate between the tubes and double‐walled tubes would be formed (Figure 8c).^147^, ^148^ Moreover, when the first layer of nanotubes is treated with an organic hydrophobic monolayer (octadecylphosphonic acid, ODPA), and re‐grown again in an organic electrolyte, amphiphilic tube stacks would be fabricated (Figure 8d).^149^

### 3.4 Template Method

**Figure 9:**
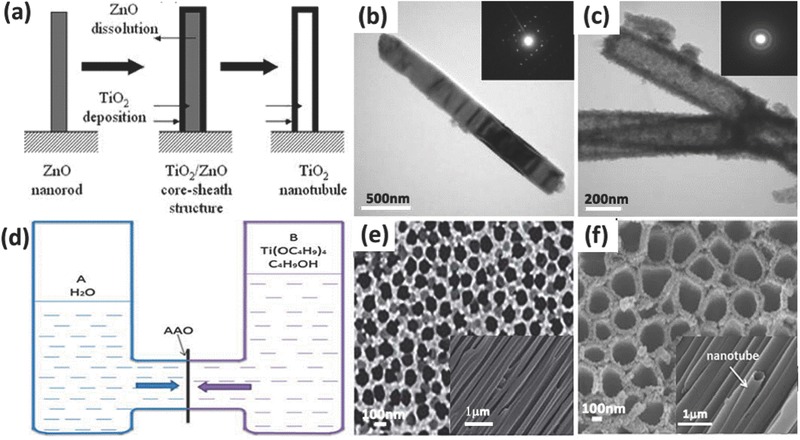
Schematic of the steps for forming the end‐closed TiO_2_ nanotube using positive template (a). TEM images of ZnO nanorods (b), TiO_2_ nanotubes (c), and their associated selected area electron diffraction patterns. Schematic diagram of the experimental device composed of two half U‐tube cells separated by an AAO membrane (d), the typical surface and section SEM images of AAO (e), and SEM images of TiO_2_ nanotubes prepared with Ti(OC_4_H_9_)_4_ solution in half‐cell B (f).(a–c) Reproduced with permission.^163^ Copyright 2005, Royal Society of Chemistry. (d–f) Reproduced with permission.^164^ Copyright 2013, Royal Society of Chemistry.

Template method is a very commonly used synthesis technique that prepares nanostructure with a morphology which follows the known and characterized templates. Typically, anodic aluminium membranes (AAM), ZnO, and silica etc. are used as templates,^150^, ^151^, ^152^, ^153^ because they can be easily removed via chemical etching or combustion, leaving the resultants with a pre‐set porosity and reversely duplicated morphology. In general, numerous TiO_2_ materials in various morphologies can be prepared easily by adjusting the morphology of the template material in certain conditions, such as TiO_2_ nanoparticles,^154^ TiO_2_ hollow fibers,^155^, ^156^ TiO_2_ spheres^157^, ^158^ and so on. Templates can be divided into positive and negative according to the way the materials grow with the templates.^159^, ^160^ Positive template synthesis leads to outer surface coating of the materials,^161^ while negative template synthesis are suitable for those to be deposited inside the template inter space.^162^ Lee et al. fabricated aligned TiO_2_ one‐dimensional nanotube arrays using a one‐step positive templating solution approach (**Figure** **9**a–c).^163^ The deposition of TiO_2_ and the selective‐etching of the ZnO template proceeded at the same time through careful control of process parameters. By precisely controlling the deposition time, the resulted different thickness of TiO_2_ sheaths lead to the formation of nanotubes or nanorods. In addition, Yuan et al. successfully developed TiO_2_ nanotube arrays through an anodic aluminium oxide (AAO) template‐based Ti(OC_4_H_9_)_4_ hydrolysis process (Figure 9d–f).^164^ In the synthesis process, two kinds of solutions in either half of the U‐tube are allowed to diffuse across the holes of the AAO membrane and then react through hydrolysis or precipitation at the interface. By carefully controlling the molar concentrations of Ti(OC_4_H_9_)_4_, TiO_2_ nanotube arrays can be obtained.

The first report on preparation of TiO_2_ nanotubes using template‐assisted method was by Hoyer et al. in 1996.^165^ In their work, TiO_2_ nanotubes were formed by electrochemical deposition into polymethyl methacrylate (PMMA) which was produced by porous AAO membrane. Zinc oxide (ZnO) nanostructure was commonly employed as a template due to its low cost and easy fabrication, and it can be easily dissolved in mild acids. As described before, Lee et al. also demonstrated that the removal of ZnO nanorod template when prepared TiO_2_ nanotubes could even be achieved by the reaction with hydrogen ions during liquid phase deposition process.^163^ Besides, carbon nanotubes have been considered to be an ideal template due to its small diameter, easy removal and self‐supported tubular morphology.^166^

**Figure 10:**
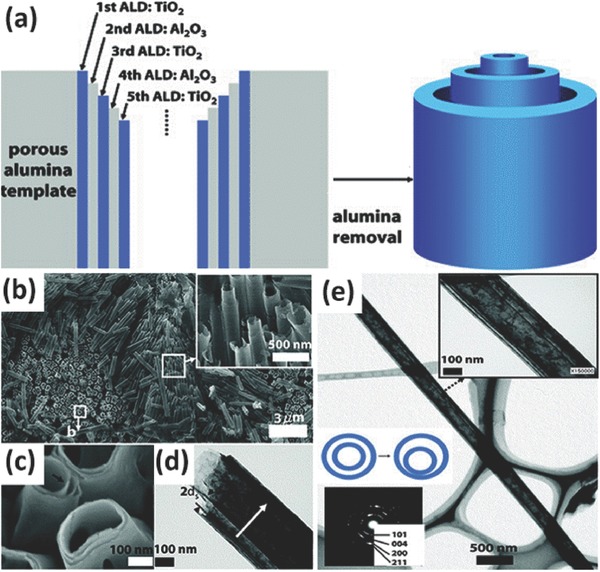
Schematic illustration of process to fabricate multiwall anatase TiO_2_ nanotubes using negative template (a). SEM image of triple‐wall TiO_2_ NTAs and sacrificial Al_2_O_3_ layers (b), and the broken ends of triple‐wall NTAs (c). TEM of a sextuple‐wall TiO_2_ NTAs (d). TEM image of long quintuple‐wall NTs: the left inset is a representative electron diffraction pattern, and the right inset shows the separated TiO_2_ walls (e). Reproduced with permission.^168^ Copyright 2009, American Chemical Society.

In a typical template preparation process, TiO_2_ sol–gel is prepared by mixing tetrabutyl titanate or titanium isopropoxide in acetic acid in the presence of templating agents. Then the polymerization of TiO_2_ in the templates or deposition of TiO_2_ onto the surface of the template aggregates occurrs. Finally, selectively removal of the templating agent and calcination of the resultants are needed.^167^ In addition, atomic layer deposition (ALD) combined template is also a good method to prepare TiO_2_ nanomaterials with certain structures. Bae et al. successfully fabricated multi‐walled anatase TiO_2_ nanotubes by alternating TiO_2_ and aluminium oxide onto porous aluminium oxide templates with ALD method, followed by etching of the sacrificial aluminium oxide (**Figure** **10**). The diameter, length, wall thickness, and wall layers of the multi‐walled TiO_2_ nanotubes can be easily adjusted.^168^

However, there are also many disadvantages which cannot be ignored about the templated method. As can be seen from the preparation process, the used template needs to be removed after synthesis in most cases, which generates waste and adds to the cost of material processing. The dissolution process may have the risk of destroying the formed TiO_2_ nanostructures. Besides, it requires tedious steps including pre‐fabrication template and post‐removal of template, which are time consuming and laborious for the practical applications. Moreover, the size and morphology varieties of templates are limited. In this regard, template method may not be suitable for large scale TiO_2_ nanotube preparation.

## 4 Surface Engineering Strategy

TiO_2_ nanostructured materials are widely used in photo­catalysis, dye‐sensitized solar cells, water splitting, lithium‐ion batteries and biomedical devices due to its low‐cost, good physical and chemical properties. As an ideal photocatalytic material, it must possess a sufficiently large specific surface area, high light absorption efficiency over a broad light absorption spectrum, as well as effective separation of the photo‐induced electron/hole pairs.^169^ However, associated with the width of energy band gap (anatase: 3.2 eV, rutile: 3.0 eV), TiO_2_ can only absorb ultraviolet light (3–5% solar light), leaving the abundant visible light from the Sun unutilized. In addition, the recombination of electron/holes is very fast, and all these disadvantages largely limit the wide applications of TiO_2_ for solar energy applications. Especially for the anodized TiO_2_ nanotube arrays, they have lower specific surface area and unconformable surface compared to TiO_2_ nanoparticles. TiO_2_ nanotubes synthesized by hydrothermal method are usually unfavorable for good contact with reactants in rigorous conditions. Therefore, it is of great importance to overcome these drawbacks to improve power conversion efficiency and photo/photoelectro‐catalytic efficiencies. Over the past decades, considerable efforts have been put into extending the light absorption, enlarging surface area and suppressing combination of electron/holes.^170^, ^171^, ^172^, ^173^, ^174^

### 4.1 Enlargement of the Photocatalytically Active Surface

**Figure 11:**
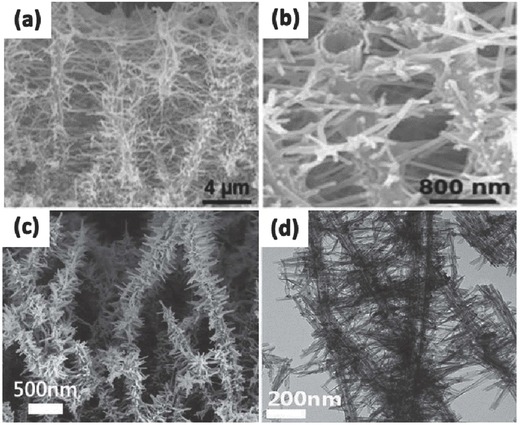
SEM images of branched double‐shelled TiO_2_ nanotubes in low (a) and high resolution (b). SEM image of hierarchical pine tree‐like TiO_2_ nanotube array (c) and its TEM image (d). (a,b) Reproduced with permission.^161^ Copyright 2012, Royal Society of Chemistry.(c,d) Reproduced with permission.^175^

It is noted the photocatalysis of 1D TiO_2_ for degradation of organic molecules or water‐splitting only occur on the surface where contains enough photocatalytically active sites. Therefore, the specific surface area and the active site concentration of a photocatalyst must be taken into account for the design and synthesis of a 1D TiO_2_ nanotubes for use in photocatalytic applications. Compared with TiO_2_ nanoparticles, the 1D TiO_2_ nanostructure has lower specific surface area and lower photocatalytic performance. To enlarge the specific surface area, there are two effective methods in general: (1) decorating second phase materials such as nanoparticles, nanorods or nanowires on the surface of the 1D TiO_2_ nanostructure; (2) constructing a coarse surface with numerous uniformly distributed nanoparticles using an acid corrosion process. Though the size, distribution and density of the loaded nanomaterials on the 1D TiO_2_ nanotubes are difficult to control, both approaches greatly improve its photocatalytic activity. On the one hand, the surface area is significantly increased due to the large specific surface area of the nanoparticles on the surface; on the other hand, nanomaterials on the 1D TiO_2_ nanostructure surface form surface heterostructures, which enhances the separation of photo‐induced charge carriers and holes. By using TiO_2_ nanotubes as backbones, branched double‐shelled TiO_2_ nanotubes can be synthesized on fluorine doped tin oxide (FTO) substrates via ZnO nanorod array template‐assisted method (**Figure** **11**a,b).^161^ The ZnO templates were removed by using wet chemical etching in 0.015 M TiCl_4_ aqueous solution for 1.5 h at room temperature. The branched nanotubes have good power conversion efficiency resulted from the increased surface area and the dye loading due to the radial TiO_2_ branches with a small diameter. In addition, Roh et al. prepared hierarchical pine tree‐like TiO_2_ nanotube arrays consisting of a vertically oriented long nanotube stem and a large number of short nanorod branches with an anatase phase directly grown on an FTO substrate via a one‐step hydrothermal process (Figure 11c,d).^175^ The morphologies of pine tree‐like TiO_2_ nanotube can be controlled by adjusting the water/diethylene glycol ratio. Owing to the larger surface area, improved electron transport and reduced electrolyte/electrode interfacial resistance from the pine tree‐like TiO_2_ nanotube arrays, the assembled dye‐sensitized solar cells exhibited a superior power conversion efficiency of 8.0%.

**Figure 12:**
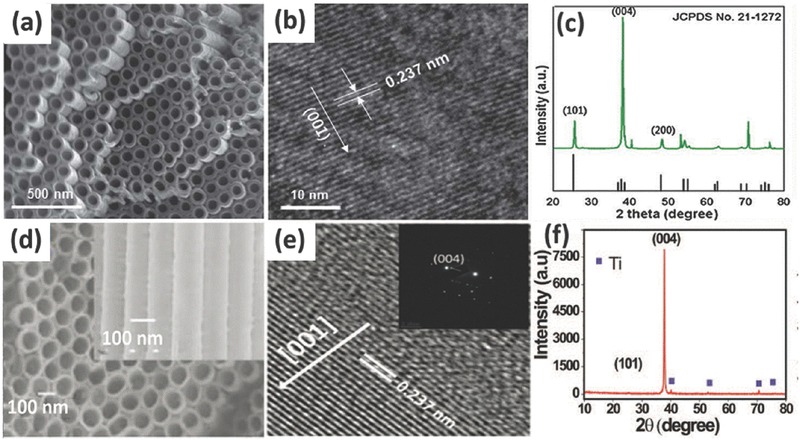
SEM (a) and TEM (b) images of a TiO_2_ nanotube array. XRD patterns of TiO_2_ nanotubes after annealing (c). SEM image (d), high resolution TEM image (e) and XRD image (f) of single crystal like Zn‐incorporated TiO_2_ nanotubes representatively. (a–c) Reproduced with permission.^183^ Copyright 2012, Royal Society of Chemistry. (d–f) Reproduced with permission.^184^ Copyright 2015, Royal Society of Chemistry.

In addition, the chemical activity of anatase TiO_2_ is closely related to its different facets which are determined by the surface energy. It is reported that the surface formation energies of TiO_2_ facets are 0.90 J m^–2^ for the (001), 0.53 J m^–2^ for the (100) and 0.44 J m^–2^ for the (101).^176^ The (001) facet possesses the highest surface energy due to the coordinatively unsaturated Ti and O atoms on (001) and very large Ti–O–Ti bond angles,^177^, ^178^ but it is difficult to prepare exposed (001) facets in TiO_2_ nanostructures due to the reduced stability, or the proposed synthesis method is time‐consuming with high cost and low efficiency. Recently, Lu et al. made great progress in the preparation of anatase TiO_2_ with exposed (001) facets using TiF_4_ as the raw material via the hydrothermal method.^179^ Inspired by this, many efforts have been put on preparing anatase TiO_2_ with exposed (001) facets from different starting chemicals since then.^180^, ^181^, ^182^ Jung et al. synthesized single‐crystal‐like anatase TiO_2_ nanotube with a mainly exposed and chemically active (001) facet relied on an oriented attachment mechanism using surfactant‐assisted processes with poly (vinyl pyrrolidone) (PVP) and acetic acid (**Figure** **12**a–c).^183^ The PVP in electrolyte was preferentially adsorbed onto the (101) surfaces, and then the growth of the (001) facets proceeded more quickly, leading to single‐crystal‐like anatase TiO_2_ with mainly the (001) plane. Such highly exposed (001) facets have shown high efficiency in retarding the occurrence of charge recombination in dye sensitized solar cells, and led to enhanced conversion efficiency. Recently, John et al. fabricated single crystal like TiO_2_ nanotubes oriented along the (001) direction with improved electronic transport property.^184^ The tubes were synthesised by a two‐stage technique: in the first stage, well‐aligned and uniform amorphous TiO_2_ nanotubes were fabricated on a titanium foil by electrochemical anodization technique; the second stage consists of zinc assisted preferential orientation of grains in the nanotubes. In this stage, Zn incorporation in the amorphous nanotubes is done in a three electrode system by applying a negative voltage to TiO_2_ nanotubes with ZnSO_4_ solution as the electrolyte. After annealing, the surface deposited oxidized zinc was removed by dipping the tubes in HCl solution; finally (001) orientated single crystal like nanotubes were obtained (Figure 12d–f). The success of this method to grow the nanotubes with the intense (001) preferential orientation could be attributed to the zinc assisted minimization of the (001) surface energy. The single crystal like TiO_2_ nanotubes shown superior electrochemical performance as supercapacitor electrodes.

### 4.2 Broadening of Light Absorption

TiO_2_ has a wide band gap of about 3.2 eV and can only absorb UV light, which only makes up 3–5% of solar light.^181^, ^185^ This intrinsic drawback largely limits the harvesting of solar light. Thus it is of great importance to enhance the absorption of the 1D TiO_2_ nanostructure by enhancement of light harvesting or broadening the light absorption from UV light to the visible light range.

**Figure 13:**
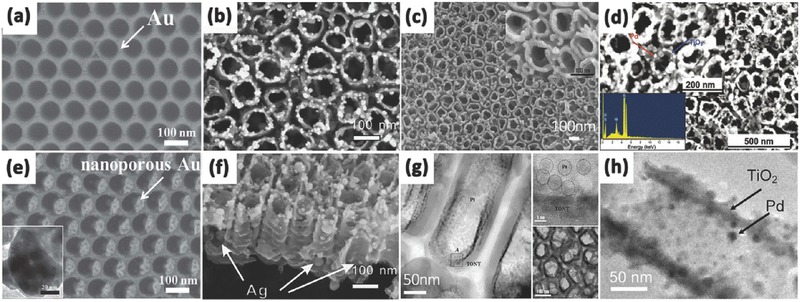
TiO_2_ nanotubes after 10 nm Au sputtering (a) and porous Au nanoparticle after dealloying in HNO_3_ (e). Top view (b) and cross‐sectional (f) SEM images of TiO_2_ nanotube arrays obtained under pulse current deposition. SEM (c) and TEM (g) images of Pt sputtered on TiO_2_ nanotube arrays. SEM (inset shows the EDX spectrum of the Pd/TiO_2_ surface) (d) and TEM (h) image of Pd nanoparticle‐functionalized TiO_2_ nanotube.(a,e) Reproduced with permission.^187^ (b,f) Reproduced with permission.^198^ Copyright 2010, Elsevier. (c,g) Reproduced with permission.^199^ Copyright 2008, The Electrochemical Society.(d,h) Reproduced with permission.^200^ Copyright 2008, American Chemical Society.

Enhancing the light absorption ability is usually realized by utilizing the surface plasmon resonance (SPR) effect of metallic nanoparticles assembled on the surface of the 1D TiO_2_ nanostructure. The decorating materials used mainly focused on various noble metals (Au, Ag, Pt, Pd or alloys) because they can be optically excited in the visible region.^186^, ^187^, ^188^, ^189^, ^190^ The modification with noble metals can also restrain the recombination of electron/hole pairs and enhance photo/photoelectro‐catalytic activity. Upon visible light illumination, the noble metal nanoparticles could be photo‐excited and generate electrons on its surface due to the SPR effect. Moreover, SPR can improve solar light conversion because of enhanced light absorption and scattering at the interface of the heterostructure and induce direct electron‐hole separation as well as plasmonic energy‐induced electron‐hole separation. All of these processes can greatly increase solar light conversion efficiency.^191^, ^192^, ^193^, ^194^, ^195^ Strategies used for decorating 1D TiO_2_ nanomaterials with noble metal nanoparticles always include UV irradiation reduction, plasma sputtering, electrodeposition, electrospinning and hydrothermal methods, among which the hydrothermal method has been most widely used due to its better control of the metal particle size and dispersion. Wu et al. constructed highly dispersed Au nanoparticles on the TiO_2_ nanotube arrays by the electrodeposition method, and the composite Au/TiO_2_ NTAs showed much higher photocatalytic degradation of methyl orange (MO) under visible light.^196^ Nguyen et al. demonstrated a novel method for fabricating a photocatalytic platform consisting of anodic TiO_2_ nanotubes supporting arrays of highly ordered porous Au nanoparticles (**Figure** **13**a,e).^187^ This approach used highly ordered‐TiO_2_ nanotubes as a morphological guide for an optimized sputtering‐dewetting‐dealloying sequential approach of a co‐catalyst layer. The metal nanoparticle size, shape, and distribution were controlled uniformly and the final nanoporous Au/TiO_2_ nanostructures showed an enhanced photocatalytic hydrogen production from ethanol/water mixtures. Similarly, Yin et al. uniformly deposited Au nanoparticles on the surface of highly ordered TiO_2_ nanotube arrays through anodization and microwave‐assisted chemical reduction route.^197^ The composite Au/TiO_2_ nanotubes exhibited excellent visible light absorption due to the localized SPR effect of Au nanoparticles. Typically, the synergistic effect between nanotubular structures of TiO_2_ and Au nanoparticles, as well as the small bias potential and strong interaction between Au and TiO_2_, facilitated the Au plasmon‐induced charge separation and transfer, which lead to highly efficient and stable photoelectrocatalytic activity. Xie et al. constructed highly dispersed Ag nanoparticles on TiO_2_ nanotube arrays by pulse current deposition (Figure 13b,f).^198^ The Ag/TiO_2_ nanotube arrays exhibited higher photocatalytic activities than the pure TiO_2_ nanotube arrays under both UV and visible light irradiation. Lee and his colleague synthesized Pt/TiO_2_ nanotube catalysts by two different deposition methods of sputtering and evaporation (Figure 13c,g).^199^ It is evident that Pt particles are deposited preferentially to the open top end of the TiO_2_ by sputtering, which results in a more conformal coating than evaporation. The Pt deposited on the nanotubes enhanced the rate of oxygen reduction reaction and it is promising for fuel cell applications. Similarly, Mohapatra et al. prepared vertically oriented TiO_2_ nanotube arrays functionalized with Pd nanoparticles of ∼10 nm size.^200^ The Pd nanoparticles distributed uniformly throughout the TiO_2_ nanotubular surface by a simple incipient wetness method (Figure 13d,h). This functionalized material was found to be an excellent heterogeneous photocatalyst for rapid and efficient decomposition of non‐biodegradable azo dyes (e.g., methyl red and methyl orange) under solar light.

It is noted that loading of metallic particles should be controlled carefully. If the amount is excessively high, the channels of TiO_2_ nanotubes could be partially blocked, leading to the decrease in the photo/photoelectro‐catalytic activity.

**Figure 14:**
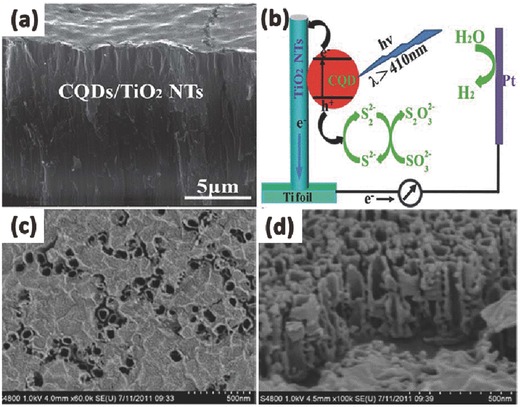
SEM images (a) of TiO_2_ nanotubes after CQD deposition. (b) The schematic diagram of the sensitization mechanism of the CQDs deposited on the surface TiO_2_ nanotubes. The top (c) and cross‐section (d) of GO/TiO_2_ composite nanotube arrays. (a,b) Reproduced with permission.^205^ Copyright 2013, Royal Society of Chemistry. (c,d) Reproduced with permission.^206^ Copyright 2012, Royal Society of Chemistry.

At the same time, broadening the photocatalytically active wavelength region to visible light is also one of the most important design principles for fabricating improved 1D TiO_2_ nanotubes. Tuning the physical and chemical properties of the TiO_2_ and constructing surface heterostructures by semiconductor materials are good choices. By modifying the high UV photocatalytic properties of TiO_2_ with the visible photocatalytic properties of semiconductor nanoparticles or specially shaped noble metal nanoparticles with a narrower band gap, an active light wavelength broadened photocatalyst can be achieved. Besides, except for UV light and visible light, infrared light comprises more than 50% of solar light energy. Thus making use of infrared light would have great potential for photocatalysis or water splitting for hydrogen generation. An important approach for assembly of TiO_2_ nanostructures with visible‐infrared light photocatalytic activity is to decorating TiO_2_ with specific materials containing d‐block or f‐block elements (such as Yb^3+^, Er^3+^, etc.), carbon or graphene quantum dots which absorbed long wavelength light and converted to shorter wavelengths that lie within the visible and UV regions.^201^, ^202^ TiO_2_ nanotubes can easily obtain high photocatalytic performance under near‐infrared light irradiation by assembling these up‐conversion nanoparticles. Since graphene or carbon quantum dots can convert infrared light to visible light, and then to UV light, it is also promising to use these newly emerging material to improve the photocatalytic properties of TiO_2_.^203^, ^204^ Zhang et al. fabricated TiO_2_ nanotube arrays loaded with carbon quantum dots (CQD) by electrodeposition (**Figure** **14**a).^205^ The excited electrons from the CQDs transferred to the conduction band of the TiO_2_ nanotubes in contact, and then transported to the counter electrode for the hydrogen evolution reaction (Figure 14b). The CQDs that were electrodeposited on the TiO_2_ nanotubes can significantly broaden the photoresponse range to the visible and NIR regions. As a result, the enhanced optical absorption can effectively improve the light‐to‐electricity efficiency for hydrogen generation. Song et al. developed a hybrid material composed of graphene oxide (GO) network on the surface of TiO_2_ nanotube arrays by a simple impregnation method.^206^ After a simple assembly process in the GO suspension, a sheet of GO coated the most surface of the TiO_2_ nanotube arrays (Figure 14c,d). The obtained GO modified TiO_2_ nanotube arrays showed a 15 times increase in the photoconversion efficiency (η) compared with pristine TiO_2_ under visible light illumination. The use of carbon and graphene quantum dots is cost‐effective, environmentally friendly, and thus promising to combine with TiO_2_ for the utilization of visible light under ambient conditions.

### 4.3 Suppression of Charge Carrier Recombination

The photocatalytic capacity of TiO_2_ mainly originates from photo‐induced charge carriers, while the charge carriers are governed by the excitation of electrons from the valence band to the conduction band, the diffusion of charge carriers and their recombination/separation that occurs at the surface or in the bulk.^169^ Thus to improve photo/electro‐catalytic water splitting efficiency, attention should be put not only on the increase of photo‐generated charge carriers by enhancement or extension of light absorption, but also on how to prohibit the recombination of these carriers.

#### 4.3.1 Heterojunction

When the TiO_2_ is coupled with SPR metal particles, electrons excited in the conduction band can escape from the plasmonic nanostructures and transfer to a contacted semiconductor, thereby forming a metal‐semiconductor Schottky junction.^207^, ^208^ These semiconductors have a great effect on the charge injection efficiency under suitable conditions. And photo‐induced charge carriers in the semiconductor can accumulate at the interface or flow into the metal leaving holes in the valence band of the semiconductor, then the charge carriers are significantly separated.

**Figure 15:**
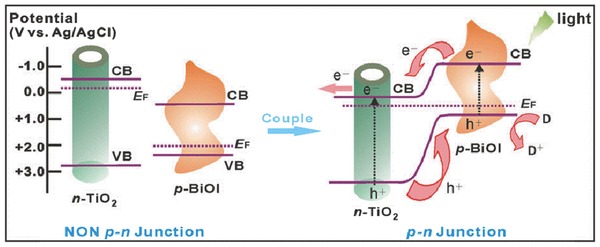
Schematic diagrams for the energy bands of p‐type BiOI and n‐type TiO_2_ before and after coupling, as well as the specific charge transfer process at the formed p‐n junction under visible‐light irradiation. Reproduced with permission.^213^ Copyright 2014, Nature Publishing Group.

Another important principle for 1D TiO_2_ nanotubes to improve photo‐induced carrier separation is to form p‐n junction between the surface nanoparticles and the 1D TiO_2_ substrate. Since TiO_2_ is an n‐type semiconductor, it would be one of the most effective strategies to construct a p‐n junction with another p‐type semiconductor due to the existence of an internal electric field at the interface.^209^, ^210^, ^211^, ^212^ When a p‐n junction is constructed, the formed local electric field drive the photo‐generated electrons move to the n‐type semiconductor side, and holes to the p‐type semiconductor side (**Figure** **15**).^213^ At the same time, the charge carriers can diffuse into the space charge region. As a result, the separation of photo‐induced electron‐hole pairs can be effectively realized, leading to enhanced photo/photoelectro‐catalytic activity.

**Figure 16:**
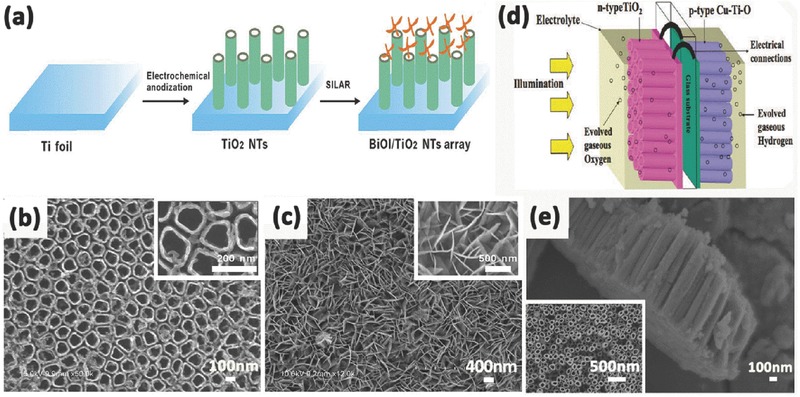
Schematic illustration for fabricating crossed BiOI nanoflakes/TiO_2_ nanotubes arrayed structure (a). SEM image of the self‐organized TiO_2_ nanotubes (b) and 3D interlaced network of BiOI layer on TiO_2_ nanotubes (c). Illustration of photoelectrochemical diode for water splitting comprised of n‐type TiO_2_ and p‐type Cu–Ti–O nanotube array films, with their substrates connected through an ohmic contact (d), lateral and top view FESEM images of Cu–Ti–O nanotube array (e). (a–c) Reproduced with permission.^213^ Copyright 2014, Nature Publishing Group. (d,e) Reproduced with permission.^215^ Copyright 2008, American Chemical Society.

Liu et al. successfully decorated the n‐type TiO_2_ with p‐type BiOI by immersing annealed TiO_2_ NTAs into Bi(NO_3_)_3_·5H_2_O and NaI, respectively.^214^ The BiOI nanoflakes grow perpendicular to the wall of nanotubes, which is beneficial for the increase in the specific surface area. And they loaded on both the outer and inner walls of TiO_2_ nanotubes, acting as the light‐transfer paths for distribution of the photo energy onto the deeper surfaces. Furthermore, the internal electric field caused by p‐n BiOI/TiO_2_ heterojunction effectively prevented the recombination of electrons and holes. Accordingly, the BiOI/TiO_2_ NTAs exhibited a more effective photo‐conversion efficiency than single TiO_2_ nanotubes under visible light irradiation. Zhao et al. used the p‐n heterojunction comprised of p‐type BiOI nanoflakes array and n‐type TiO_2_ nanotubes array fabricated by successive ionic layer adsorption and reaction (SILAR) (**Figure** **16**a) for the detection of cancer biomarker vascular endothelial growth factor.^213^ The crossed BiOI nanoflakes on perpendicularly aligned and highly ordered TiO_2_ nanotubes possessed unique layered structures (Figure 16b,c). Specifically, charge separation could occur simultaneously both in BiOI nanoflakes and TiO_2_ NTAs. Then, the photoelectrons of p‐type BiOI would promptly inject into the conuction of n‐type TiO_2_, while the holes of latter would transfer to the valence band of the former. These injected electrons on the conduction band of TiO_2_ NTAs were then rapidly collected by the Ti substrate as photocurrent due to the efficient charge transport within the arrayed tubes. The synergistic effect of these factors could substantially promote the spatial charge separation and the subsequent migration of these carriers, impeding the charge recombination and thus improving the excitation and conversion efficiency. Mor et al. fabricated vertically oriented p‐type Cu–Ti–O nanotube array films by anodization of copper rich (60% to 74%) Ti metal films co‐sputtered onto FTO coated glass (Figure 16e).^215^ In combination with n‐type TiO_2_ nanotube array films, p/n‐junction photochemical diodes capable of generating a chemical fuel were formed (Figure 16d) with a photocurrent of approximately 0.25 mA cm^2^, at a photoconversion efficiency of 0.30%.

**Figure 17:**
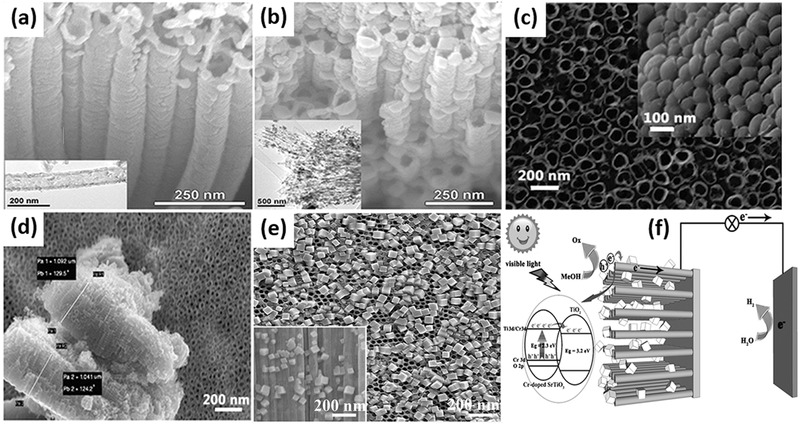
SEM and TEM (insets) images of (a) TiO_2_ nanotube array after annealing at 450 °C and (b) TiO_2_–SrTiO_3_ hybrid nanostructures obtained after 1 h hydrothermal treatment. Top‐view and bottom‐view (inset) images of Sr‐doped TiO_2_ nanotubes (c) and its corresponding cross‐sectional views in 0.04 M dopant concentration at pH = 3 (d). SEM images (e) and schematic illustration (f) of charge separation and transport of heterostructured Cr‐doped SrTiO_3_ nanocube/TiO_2_ nanotube array heterostructures. (a,b) Reproduced with permission.^220^ Copyright 2009, American Chemical Society. (c,d) Reproduced with permission.^221^ Copyright 2011, American Chemical Society. (e,f) Reproduced with permission.^223^

Some ternary metal oxides with the perovskite structure such as SrTiO_3_, CaTiO_3_, BaTiO_3_, MgTiO_3_, etc., have been found to be catalytically active and can be potentially used in photo­catalysis by forming TiO_2_ heterostructures.^48^, ^216^, ^217^, ^218^, ^219^ Of particular interest, SrTiO_3_ has attracted much interest for water splitting due to its high corrosion resistance and excellent photocatalytic activity. Furthermore, SrTiO_3_ possesses only a 200 mV conduction band edge, which is more negative than TiO_2_, thus it makes SrTiO_3_ a good candidate for coupling TiO_2_ and improving photoelectrochemical properties by shifting the Fermi level of the composite to more negative potentials.^220^ In 2009, Zhang et al. tailored TiO_2_–SrTiO_3_ heterostructure nanotube arrays by converting electrochemical anodized TiO_2_ nanotube arrays into TiO_2_–SrTiO_3_ heterostructures through controlled substitution of Sr under hydrothermal conditions (**Figure** **17**a,b).^220^ The photoelectrochemical performance of such a vertically aligned heterostructure array is strongly dependent on its composition and morphology. At the hydrothermal reaction time equal to 1 h, nanoparticles with a diameter of approximately 50 nm start to form on the surface of TiO_2_ nanotubes. In this condition the TiO_2_ nanotube electrodes exhibited greater photocurrent than other composite TiO_2_ nanotubes with longer reacting time. Only well‐dispersed SrTiO_3_ nanocrystallites on TiO_2_ nanotube arrays can efficiently cause the Fermi level to equilibrate and reduce the recombination of charge carriers at the surface of the heterostructure, and finally improve the overall photoelectrochemical performance. This work provides a convenient way to tailor the photoelectrochemical properties of TiO_2_–SrTiO_3_ nanotube arrays and employ them for dye‐sensitized solar cells or photocatalytic hydrogen production. In 2011, Hamedani et al. reported highly ordered Sr‐doped TiO_2_ nanotube arrays synthesized via a one‐step electrochemical anodization technique (Figure 17c,d).^221^ The morphology and quality of the fabricated materials were highly related to the pH of the electrolyte and the solubility limit of Sr(OH)_2_ in the electrolyte. Moreover, Sr doping of TiO_2_ nanotubes showed a red shift in the absorption edge, which resulted in an electrode photoconversion efficiency of 0.69%, more than 3 times higher than that of the undoped nanotube arrays (0.2%) under the same conditions. Additionally, metallic elements such as Nb, Cr are usually doped into TiO_2_/SrTiO_3_ nanotubular heterostructures to enhance their photoelectrocatalytic activities.^222^, ^223^ For example in the SrTiO_3_/TiO_2_ composites, the electrons of pure SrTiO_3_ can only be excited from the valence band (O 2p) to the conduction band (Ti 3d) by photons with energy greater than 3.2 eV. While in Cr‐doped SrTiO_3_, a Cr 3d level appears within the forbidden gap, and the mixing of the Cr 3d with the Ti 3d level slightly lowers the conduction band, which effectively narrows the bandgap of SrTiO_3_. So electrons can directly transit from the newly formed dopant states (Cr 3d) to the conduction band (Ti 3d and Cr 3d). In this way the visible‐light response of SrTiO_3_/TiO_2_ heterostructures can be greatly improved by the partial substitution of Cr^3+^ for Sr^2+^ cations in the SrTiO_3_ (Figure 17e,f).^223^

#### 4.3.2 Band Structure Engineering

A good matching of the conduction and valence bands of two semiconductors enables efficient charge carrier transfer from one to another.^224^ It is reported that wide band gap energy of TiO_2_ nanotubes coupled with small band gap semiconductor could result in the formation of heterojunctions, which simultaneous enhances visible light harvest and charge separation.^225^, ^226^

**Figure 18:**
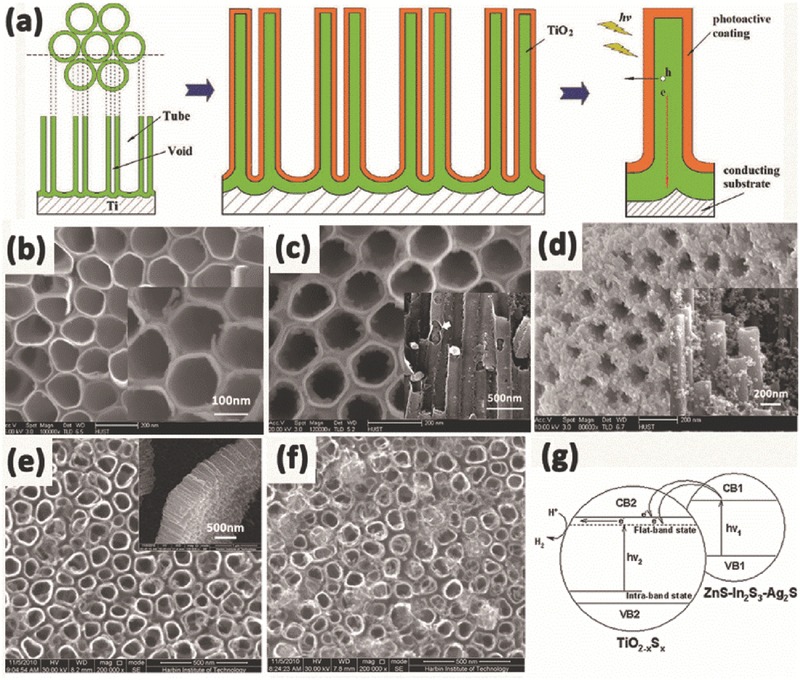
Schematic of narrow‐band‐gap semiconductor/TiO_2_ nanotube coaxial heterogeneous structural design (a). Top‐surface FE‐SEM and enlarged (inset) images of CdS electrodeposited onto TiO_2_ nanotube arrays at –0.65 V (b), –0.70 V (c) and –0.75 V (d) (vs Ag/AgCl) representatively. SEM images of TiO_2_ nanotubes (e) and ZnS–In_2_S_3_–Ag_2_S@TiO_2_ nanotubes (f). The band structures and the mechanism of electron transport for ZnS–In_2_S_3_–Ag_2_S solid solution coupled with TiO_2–x_S_x_ nanotubes film catalyst (g). (a–d) Reproduced with permission.^229^ Copyright 2010, American Chemical Society. (e–g) Reproduced with permission.^234^ Copyright 2012, Elsevier.

The modification of TiO_2_ nanotubes with quantum dots with narrow band gap is a promising approach since it can improve the photocatalytic activity effectively. Cadmium sulfide (CdS) is a well‐known semiconductor and widely used to modify TiO_2_ materials. CdS has a narrow band gap of 2.4 eV, which matches well with the spectrum of sunlight and allows CdS to act as photo‐sensitizer to absorb visible light. The combination of CdS and TiO_2_ can be achieved by electrochemical deposition and sequential chemical bath deposition. In a typical synthesis procedure, dipping the prepared TiO_2_ nanotube arrays film into the precursor solution containing Cd^2+^ for several minutes and dried at 60 °C in air are needed first. Then them are placed into the precursor solution of S^2–^ followed by treatment with electrochemical deposition or sequential chemical bath deposition. Usually the two steps are repeated several times to realize a total deposition. And finally the mixtures are heated at 450 °C for 2 h in a nitrogen atmosphere.^227^, ^228^ Zhu et al. reported the coaxial heterogeneous composite material formed from self‐assembled TiO_2_ nanotubes and the narrow‐band‐gap compound semiconductor CdS using the electrochemical ALD technique (**Figure** **18**a).^229^ The modified electrochemical ALD technique produced uniform thin films of narrow‐band‐gap compound semiconductors coating onto large‐surface‐area nanostructured substrates in various deposition potentials (Figure 18b–d). These coaxial heterogeneous structures enhanced CdS/TiO_2_ and CdS/electrolyte contact areas and reduced the distance of holes and electrons to the electrolyte or underlying conducting substrate. This results in enhanced photon absorption and photocurrent generation, which are favorable for water photoelectrolysis and toxic pollutant photocatalytic degradation. By using a small applied potential and white light, CdS‐coated TiO_2_ nanotube arrays could totally inactivate bacteria in very short time due to the enhanced generation of OH• radicals and with H^+^, which has great potential in wastewater treatment.^230^ It has been proven that TiO_2_ nanotubes have directionality benefits in oriented arrays through improvements in electron mobility and separation of charges, but the accessiblity of two sides for illumination is still limited when directly used for solar cell.^231^, ^232^ Thus the detachment and reassembly of TiO_2_ nanotubes on another conducting surface are necessary. In order to deal with such problem and assess the behavior of randomly structured nanotubes versus oriented nanotubes, David et al. conducted a photoelectrochemical study by modifying each of these systems with CdS.^233^ They removed etched TiO_2_ nanotubes from the titanium foil substrate by sonication and reassembled them onto new electrodes. The random nanotube structures can directly transfer electrons conducting substrate without a barrier layer and have a higher intertube porosity, providing additional space for the electrolyte solution, which allows for faster mass transfer of the redox couple. The photoelectrochemical results of these reassembled TiO_2_–CdS electrodes showed a slight decrease in photocurrent response but a small increase in photopotential as compared to oriented TiO_2_–CdS electrodes. Reconstruction of the nanotubes onto new substrates does not significantly reduce the benefits of the one dimensional TiO_2_ nanostructure, and it creates opportunities for various critical applications.

Besides CdS quantum dot, Jia et al. successfully prepared ZnS–In_2_S_3_–Ag_2_S solid solution coupled with TiO_2–x_S_x_ nanotubes film catalyst by a two‐step process of anodization and solvothermal methods.^234^ After sovolthermal treatment, the TiO_2_ nanotubes still kept their tube‐like structures, and the needle‐like ZnS–In_2_S_3_–Ag_2_S nanorods deposited on the most part of the surface of TiO_2_ nanotubes (Figure 18e,f). Typically, the doping of multiple elements could modify the band structure, narrowing the band gap of TiO_2_ and inducing visible light absorption at the sub‐band‐gap energies (Figure 18g). Such ZnS–In_2_S_3_–Ag_2_S@TiO_2–x_S_x_ nanotubes composite presents the enhanced absorption in visible region and the efficient transfer of photoelectron between the solid solution and TiO_2–x_S_x_ nanotubes, which leads to the excellent photocatalytic activity for the photocatalytic hydrogen evolution from aqueous solutions.

It is noted that the band gap of TiO_2_ can be narrowed by non‐metal ion doping via using anions such as N, C, B, F, P, S and I etc.^235^, ^236^, ^237^, ^238^ The commonly used methods to prepare doped TiO_2_ nanostructures are: thermal treatments or synthesis of TiO_2_ NTAs in certain gas atmospheres such as N_2_, CO, Ar, etc.; co‐sputtering or sputtering with doping materials; ion implantation and electrochemical oxidation.^147^ Thermal treatment in gas atmospheres of the doping species is frequently used for nitrogen or carbon doping. Ion implantation and electrochemical oxidation are recognized as facile doping means to incorporate nitrogen‐containing species into the TiO_2_ lattice. However, the sputtering and ion implantation need high energy accelerators in high operating voltage, and the implantation depth is only several micrometers, which lead to inhomogeneous dopant distribution. Among all the nonmetal doped TiO_2_, N‐ and C‐ doped TiO_2_ are the most successful approaches and have been most widely studied. And combing with the intrinsic defects of TiO_2_, such as reduced Ti species and oxygen vacancies, the formation of localized states which may merge to form sub‐band gap level and thus creating a lower energy excitation pathway.^105^ Recently, Su et al. synthesized a graphitic carbon nitride quantum dots (CNQDs) modified TiO_2_ nanotube arrays (NTAs) photoelectrode by electrochemical anodization technique and a followed organic molecular linkage using bifunctional organic molecule as an effective linker.^239^ The modification of TiO_2_ by CNQDs showed a significant improvement in the photoelectrochemical activity owing to enhanced light absorption and improved photo‐generated electron‐hole pairs separation. Upon solar light irradiation, photo‐generated holes and electrons are generated in the valence band (VB) and conduction band (CB) of TiO_2_ and CNQDs, respectively. These electrons in CB of CNQDs can transfer easily to the CB of TiO_2_ due to the band alignment and potential difference, and they finally reach the Ti substrate along the vertical tubular structures, realizing fast charge transfer and separation. Under the electric field, the electrons transfer to the counter electrode to complete hydrogen production. At the same time, the accumulated holes in the VB of CNQDs which come from VB of TiO_2_ continuely oxidize water to form oxygen. Thus, the photo‐generated electron‐hole pairs are effectively separated, thereby leading to the significantly enhanced photoelectrochemical performance.

## 5 Application of Photo/photoelectro‐catalytic Water Splitting

With the fast development of economy and increasing depletion of natural fossil fuels, the environmental problems and energy shortage are becoming more and more serious. Human beings have been urgently called up to deal with these problems. As discussed above, TiO_2_ nanotubes have shown to be an excellent photocatalyst for hydrogen production and decomposition of pollutants due to low‐cost, non‐toxicity, strong redox power, and physical and chemical stability.^240^, ^241^, ^242^

### 5.1 Photocatalytic Water Splitting for Hydrogen Production

**Figure 19:**
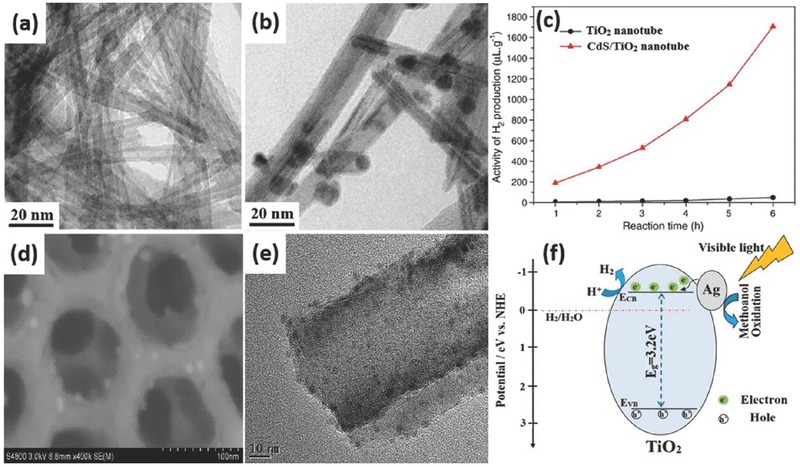
TEM images of TiO_2_ nanotube (a) and CdS/TiO_2_ nanotube (b), respectively. Activities of H_2_ evolution for TiO_2_ nanotube and CdS/nanotube under visible light irradiation for 6 h (c). SEM (d) and TEM (e) image of Ag/TiO_2_ NTAs, respectively. Schematic diagram showing the energy band structure and electron‐hole pairs separation in Ag/TiO_2_ NTAs under visible light irradiation (f). (a–c) Reproduced with permission.^251^ Copyright 2008, Elsevier. (d–f) Reproduced with permission.^256^ Copyright 2016, Royal Society of Chemistry.

Photocatalysis is the simplest water‐splitting approach, more amenable to cheap, large scale applications of H_2_ production.^243^, ^244^, ^245^ In general, the hydrogen production rate is greatly depending on the sacrificial agent (methanol, ethanol, KOH etc.), light intensity, and TiO_2_ morphology and structure.^246^ D'Elia et al. compared the hydrogen production activity of TiO_2_ nanotube with TiO_2_ nanoparticles by using methanol as sacrificial agent. TiO_2_ nanotube was found to be more active than TiO_2_ nanoparticles under UV light because of the oriented structure for superior charge transport and lower recombination of electron/hole pairs.^247^ In addition, Kim et al. and Sun et al. explored the influence of anodization time and annealing temperature of TiO_2_ nanotube arrays on the photocatalytic water splitting activity, respectively. The results showed that TiO_2_ NTAs with high ratio of anatase and media tube length displayed higher photocatalytic hydrogen production activity.^248^, ^249^ Besides, Xu et al. successfully synthesized 1D mesoporous TiO_2_ nanotubes with lengths ranging from 100 nm to 400 nm and diameters around 10 nm by a hydrothermal‐calcination process.^250^ They exhibited excellent photocatalytic activity for simultaneous photocatalytic H_2_ production and Cu^2+^ removal from water. However, TiO_2_ nanotubes still show low photocatalytic hydrogen production activity under visible light due to fast recombination of electron/hole pairs and low utilization of visible light. Therefore, it is essential to increase the surface area or construct heterostructures by modifying TiO_2_ nanotubes with metal, non‐metal and semiconductors to improve the photocatalytic performances. CdS, a well‐known semiconductor (2.4 eV), which can increase visible light absorption of TiO_2_ nanotube and facilitate the transfer of the photo‐generated electrons at the interface between CdS and TiO_2_, is widely used to modify TiO_2_ nanotubes to improve photocatalytic water splitting activity. Zhang et al. prepared CdS/TiO_2_ nanotubes composite by a facile chemical bath deposition method (**Figure** **19**a–c).^251^ Hexagonal phase CdS nanoparticles with an average particle size of ca. 8 nm were uniformly anchored inside TiO_2_ nanotubes in average tubular diameter of ca. 15 nm. The absorption edge of TiO_2_ nanotubes can be extended to visible region by loading with CdS nanoparticles. The CdS/TiO_2_ nanotubes composite exhibited high activity of hydrogen production (284.7 μL h^–1^ g^–1^) in a mixure of Na_2_S and Na_2_SO_3_ solution under visible light irradiation due to the enhanced separation efficiency of photogenerated electron‐hole pair. In addition, noble metal particles such as Au, Ag, Pt etc. are also widely used to decorate TiO_2_ nanotubes for improving photocatalytic hydrogen production activity because of surface plasmon resonance (SPR) effect for enhanced visible light absorption and suppressed combination of electron/holes.^252^, ^253^, ^254^, ^255^ Recently, we reported an enhanced hydrogen generation rate of 2 μmol h^–1^ cm^–2^ by a two‐step electrochemical anodized TiO_2_ NTAs with a ordered hexagonal and regular porous top layer.^256^ Moreover, we found that Ag nanoparticles sensitized TiO_2_ NTAs by an ultrasonication‐assisted in situ deposition strategy exhibited highly efficient photocatalytic hydrogen production rate of 30 μmol h^–1^ cm^–2^ under visible light illumination (15 times over its pristine TiO_2_ NTA counterpart) (Figure 19d–f). Except for binary, ternary Fe/Ag/TiO_2_ NTAs, Cu_2_O/Cu/TiO_2_ NTAs, CdSe/CdS/TiO_2_ NTAs structures are widely investigated and they all showed improved photocatalytic water splitting activity under both UV and visible light.^257^, ^258^, ^259^

### 5.2 Photoelectrocatalytic Water Splitting for Hydrogen Production

**Figure 20:**
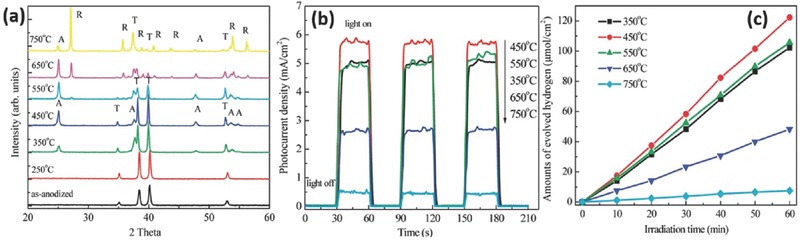
X‐ray diffraction patterns (a), transient photocurrent response (b) and comparison of the rates of hydrogen production (c) of TiO_2_ NTAs annealed at various temperatures ranging from 350 to 750 °C. A, anatase; R, rutile; T, titanium. Reproduced with permission.^266^ Copyright 2011, American Chemical Society.

Although intense efforts have been put into photocatalytic water splitting in order to increase hydrogen generation activity, it still faces several challenging issues such as fast recombination of electron/hole pairs, low quantum efficiency in the visible range and solar‐to‐hydrogen (STH) efficiencies less than 0.1%. Photoelectrolysis using photocatalyst electrodes with additional electrical power provided by a photovoltaic element has been proven a facile and promising route to improve the water splitting performance with higher STH efficiency over than 5%.^260^, ^261^, ^262^, ^263^, ^264^ Lin et al. obtained an enhanced hydrogen production by photoelectrocatalytic water splitting using extremely highly ordered nanotubular TiO_2_ arrays with three‐step electrochemical anodization.^265^ The TiO_2_ NTAs constructed through the third anodization showed appreciably more regular architecture than that of the sample by conventional single anodization under the same conditions. It was found that the photoelectrocatalytic water splitting for hydrogen evolution rate of the third‐step anodic TiO_2_ nanotubes was higher than that of single‐step and second‐step anodic TiO_2_ nanotubes. In addition, Sun's group discussed the effect of annealing temperature on the hydrogen production of ordered TiO_2_ nanotube arrays, which were synthesized by a rapid anodization process in ethylene glycol electrolyte (**Figure** **20**).^266^ The results indicated that the crystal phase and morphology of TiO_2_ NTAs had no great changes at low annealing temperatures. Anatase phase and tubular structure of TiO_2_ NTAs were stable up to 450 °C. With further increase in temperature, the crystallization transformation from anatase to rutile phase appeared, accompanied by the destruction of tubular structures. Due to the excellent crystallization and the maintenance of tubular structures, TiO_2_ NTAs annealed at 450 °C exhibited the highest photoconversion efficiency of 4.49% and maximum hydrogen production rate of 122 μmol h^–1^ cm^–2^, which is consistent with Li's results.^267^ Besides, Liang's group reported a study to improve the solar water splitting activity of TiO_2_ photoanodes by tuning the average wall thickness, inner diameter and porosity of the nanotube arrays.^268^ Further analysis reveals that the photoconversion efficiency increases monotonously with porosity rather than with wall thickness or inner diameter because large porosity can ensure a much shorter hole diffusion path toward wall surface and accelerate ion migration in the tube to overcome the kinetic bottleneck, thus enhancing the photoelectrocatalytic water splitting efficiency.

**Figure 21:**
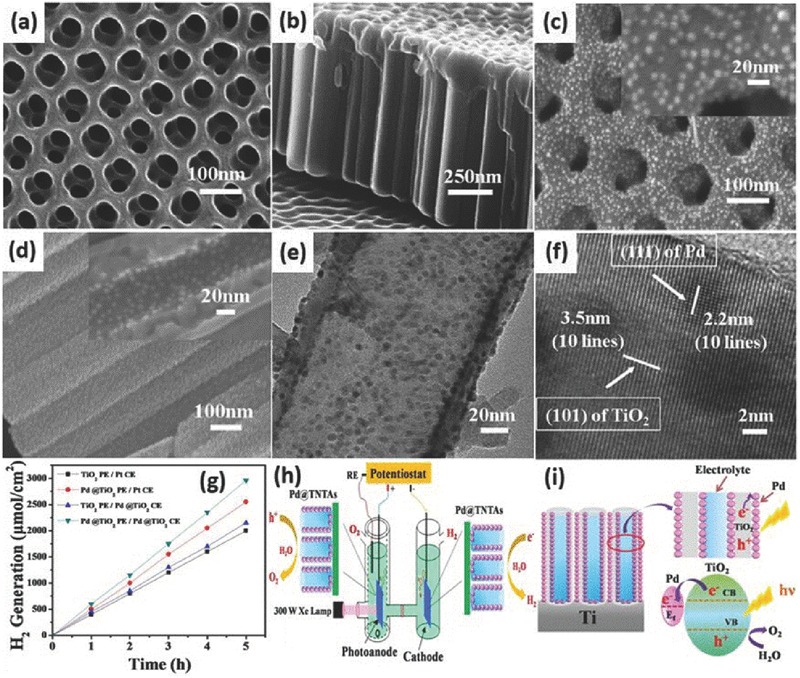
Top and cross‐sectional SEM images of pure TNAs (a,b) and Pd QDs/TNAs (c,d). The insets show the corresponding magnified images. TEM (e) and HRTEM (f) images of TNAs coated with Pd QDs. Amount of hydrogen generated by capitalizing on TiO_2_ nanotubes and Pd/TNTAs nanocomposites as photoanodes and Pt foil and Pd/TNTA nanocomposites as cathodes at –0.3 V_SCE_ in a PEC cell containing a 2 M Na_2_CO_3_ and 0.5 M ethylene glycol solution under 320 mW cm^–2^ irradiation (g). Schematic of Pd/TNAs on photoelectrolytic water splitting for hydrogen production (h). Schematic illustration of TNAs deposited with Pd QDs and the charge transfer process from TiO_2_ to Pd (i). Reproduced with permission.^135^ Copyright 2012, American Chemical Society.

However, pristine TiO_2_ nanotubes suffer from not only low electrical conductivity, but also fast recombination of electro/hole pairs and weak visible‐light harvest due to the larger band gap, resulting in a low STH conversion efficiency. The band engineering of TiO_2_ nanotubes by using dye, quantum dot sensitization, chemical doping and narrow gap semiconductor coupling has been proposed to enhance light absorption and obtain a higher STH efficiency.^269^, ^270^, ^271^ Recently, black titania was reported by annealing in the hydrogen atmosphere to boost solar light harvesting impressively for enhanced photocatalytic and photoelectrochemical performances.^272^, ^273^ This was attributed to considerable amount of Ti^3+^ defects and oxygen vacancies introduced into lattice of TiO_2_ nanotube for expanding visible light absorption, facilitating charge transport and charge separation. In addition, Ye et al. sensitized TiO_2_ nanotube arrays by palladium quantum dots (Pd QDs) by a facile hydrothermal strategy. As shown in **Figure** **21**a,b, the nanotube arrays were crack‐free and smooth with an average tube diameter of 80 nm and a wall thickness of 30 nm after a three‐step electrochemical anodization.^135^ After hydrothermal reaction, Pd QDs were uniformly dispersed over the entire surface of the nanotubes, both inside and outside of the nanotubes with very small particle size of 3.3 ± 0.7 nm (Figure 21c–f). By exploiting Pd@TNTAs nanocomposites as both photoanode and cathode, a substantially increased photon‐to‐current conversion efficiency of nearly 100% at λ = 330 nm and a greatly promoted photocatalytic hydrogen production rate of 592 μmol h^–1^ cm^–2^ at –0.3 V_SCE_ in Na_2_CO_3_ and ethylene glycol solution under 320 mW cm^–2^ irradiation were achieved (Figure 21g–i).

**Figure 22:**
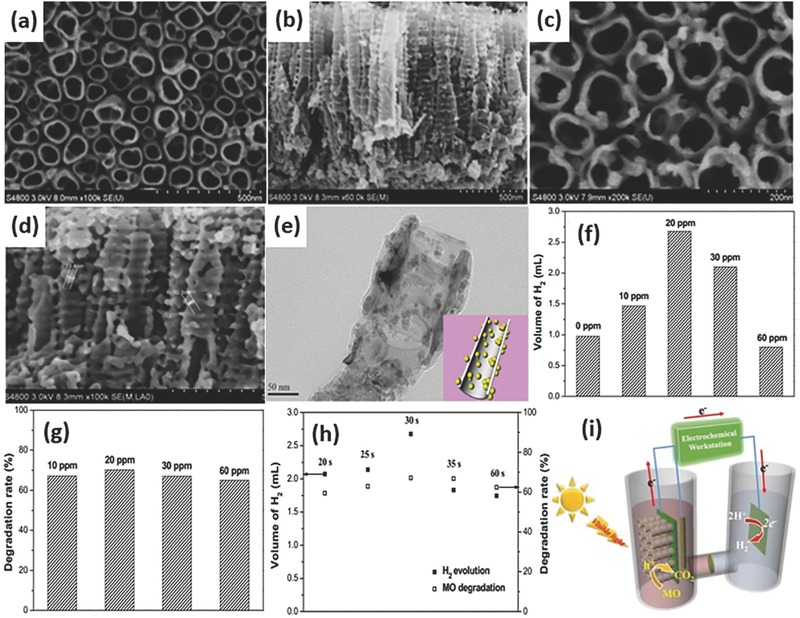
SEM images of pure TiO_2_ nanotube arrays (a,b) and CdSe/NTAs (c,d). TEM image of CdSe/NTAs (e). The volume of the evolved hydrogen on Pt foil (f), and the degradation rate of MO with different concentration MO (g) under the visible light illumination. (CdSe/TiO_2_ NTAs with electrodeposition for 30 s as photoanode, Pt foil as counter electrode, reaction time = 2 h). Photoelectrocatalytic H_2_ evolution and MO (20 ppm) degradation rates on different samples by changing the electrodeposition time (h). Schematic of CdSe/NTAs on photoelectrolytic water splitting for hydrogen production and degradation rate of MO at the same time (i). Reproduced with permission.^276^ Copyright 2016, Elsevier.

Gao et al. successfully constructed heterostructures by incorporating CdTe QDs into TiO_2_ nanotubes.^274^ It was found that CdTe QDs have significantly extended the photon response of the TiO_2_ nanotube film electrodes into the visible region and the photoelectrochemical performance of the QDs‐sensitized TiO_2_ photoelectrode was affected significantly by the size of the CdTe QDs. The photocurrent and hydrogen production activity have been improved a lot under AM 1.5 light illumination than the plain TiO_2_ nanotube film. These results confirmed that semiconductors such CdTe, CdS, Cu_2_O etc. can be used as effective sensitizers and demonstrates the potential applications of the TiO_2_ nanotube/semiconductor heterostrutures in solar cells. In order to achieve more superior hydrogen production rate, visible‐light‐driven responsive Au/reduced graphene oxide/hydrogenated TiO_2_ nanotube arrays ternary composites (Au/RGO/H‐NTAs) were fabricated by electrophoretical deposition of Au nanoparticles and graphene oxide sheets onto hydrogenated TiO_2_ nanotube arrays.^275^ Under visible light illumination (λ > 400 nm), the photoelectrochemical current density and hydrogen evolution rate of Au/RGO/H‐NTAs is 224 μA cm^–2^ and 45 μmol h^–1^ cm^–2^, which is much higher than that of pristine NTAs, single H‐NTAs or RGO/H‐NTAs due to the SPR effect of noble nanoparticles for visible light harvesting and the efficient separation of electron/hole pairs. Other than photoelectrocatalytic water splitting for hydrogen generation, photocatalysts can also degrade pollutants at the same time. CdSe nanoparticles enhanced TiO_2_ nanotube arrays electrodes (CdSe/NTAs) by electrodeposition were explored as the photoanode for driving the photoelectrocatalytic generation of hydrogen and simultaneous degradation of organic pollutants in a photoelectrochemical (PEC) system.^276^ As shown in **Figure** **22**, CdSe nanoparticles, with an average size about 10 nm, were highly dispersed and uniformly deposited on the surface of the pore wall and inside of TiO_2_ NTAs. And the amount of nanoparticles can be adjusted by the electrodeposition time. Besides, both the electrodeposition time and concentration of MO was investigated for evaluating the photoelectrocatalytic activity of CdSe/NTAs photoanode. It was also found that the presence of the MO greatly enhanced the PEC efficiency for hydrogen evolution as well as the high simultaneous degradation rates of MO. CdSe/NTAs photoanode showed high degradation activity of MO and hydrogen production rate at 0.3 bias under visible light irradiation. It was noted that deposition time for 30 s and 20 ppm concentration of MO were favorable for the highest hydrogen evolution and photoelectrocatalytic degradation. The strategy could provide experience for designing new nanocomposites that generation of hydrogen and simultaneous degradation of organic pollutant, which presents both enery and environmental benefits.

Furthermore, functionalizing TiO_2_ with biologically active materials to achieve light‐assisted water splitting is a brand new avenue to pursue. Nageh et al. have successfully prepared a bacteriorhodopsin (bR)/TiO_2_ nanotube array hybrid electrode system by the sensitization of anodized TiO_2_ nanotubes with bR.^277^ The bR/TiO_2_ electrodes anchored with a linker showed a approximately 50% increase in photocurrent density compared to pure TiO_2_ when used as photoanodes to split water photoelectrochemically. Such enhanced photocurrent generation is due to the proton pumping effect of bR, and this work may provide a new perspective method for developing versatile bio‐photoelectric devices for solar‐to‐fuel generation.

Similar to photocatalytic water splitting for hydrogen production, besides the sacrificial agent (methanol, ethanol, KOH etc.), light intensity, TiO_2_ morphology and structure, the bias potential also affects the hydrogen production rate. The comparison between photo/photoelectro‐catalytic water splitting is summarized in **Table** **2** and **Table** **3**, respectively.

**Table 2:** Summary of TiO_2_ nanotubes based materials in photocatalytic water splitting

Photocatalyst	Light intensity	Electrolyte	Water splitting rate	Ref.
TiO_2_ nanotubes	150 W halide lamp	10 v% MeOH + 90 v% H_2_O	0.27 μmol min^–1^	247
TiO_2_ nanotubes	400 W Hg lamp	MeOH + H_2_O	15.7 mmol g^–1^ h^–1^	250
ZnS–In_2_S_3_–Ag_2_S/TiO_2_ NTAs	500 W Xe lamp	0.1 M Na_2_S + 0.02 M Na_2_SO_3_	25.02 μmol h^–1^	234
CdS/TiO_2_ nanotubes	Visible light (λ > 400 nm)	Na_2_S + Na_2_SO_3_	284.7 μL g^–1^ h^–1^	251
Au/TiO_2_ nanotubes	100 mW cm^–2^ (λ > 400 nm)	10 v% MeOH + 90 v% H_2_O	482 μmol g^–1^ h^–1^	252
Pt/TiO_2_ nanotubes	Visible light (λ > 400 nm)	MeOH + H_2_O	29.2 μmol g^–1^ h^–1^	254
Pt/TiO_2_ nanotubes	120 W Hg lamp	10 v% EtOH + 90 v% H_2_O	30 mmol g^–1^ h^–1^	255
Ag/TiO_2_ NTAs	Visible light (λ > 420 nm)	20 v% MeOH + 80 v% H_2_O	30 μmol cm^–1^ h^–1^	256
Cu/Cu_2_O/TiO_2_ nanotubes	350 W Hg lamp	10 v% MeOH + 90 v% H_2_O	7.6 μmol cm^–1^ h^–1^	258
Fe/Ag/TiO_2_ nanotubes	Visible light (λ > 400 nm)	10 v% EtOH + 90 v% H_2_O	1.35 μmol cm^–2^ h^–1^	259
Pt/TiO_2_ NTAs	Solar light AM 1.5	50% MeOH + 50% H_2_O	25 μmol cm^–2^ h^–1^	278
Au/TiO_2_ nanotubes	6.5 mW cm^–2^ UV light	20 v% EtOH + 80 v% H_2_O	31.8 mmol g^–1^ h^–1^	279
C/TiO_2_ nanotube/Carbon nanotubes	Solar light AM 1.5	50 v% EtOH + 50 v% H_2_O	37.6 mmol g^–1^ h^–1^	280
Cu(OH)_2_/TiO_2_ NTAs	300 W Xe lamp	0.09 M EG + H_2_O	6.5 μmol cm^–2^ h^–1^	281
Pt/N/TiO_2_ nanotubes	250 W Hg lamp	C_3_H_8_O_3_ + H_2_O	1508 μmol g^–1^ h^–1^	282
Ag/TiO_2_ NTAs	35 mW cm^–2^ (λ > 400 nm)	10 v% EtOH + 90 v% H_2_O	0.96 μmol cm^–1^ h^–1^	283
Cu(OH)_2_/TiO_2_ NTAs	400 W Hg lamp	10 v% MeOH + 90 v% H_2_O	14.94 mmol cm^–1^ h^–1^	284
Zn/TiO_2_ nanotubes	18 W cm^–2^ UV light	MeOH + H_2_O	2.3 mL g^–1^ h^–1^	285
CuO/TiO_2_ nanotubes	400 W Hg lamp	10 v% MeOH + 90 v% H_2_O	71.6 mmol g^–1^ h^–1^	286
Pt/CdS/TiO_2_ nanotubes	500 W Hg lamp	0.1 M Na_2_S + 0.35 M Na_2_SO_3_	74 mL g^–1^ h^–1^	287
CdS/TiO_2_ nanotubes	Visible light (λ > 420 nm)	0.25 M Na_2_S + 0.35 M Na_2_SO_3_	2.62 mmol g^–1^ h^–1^	288

**Table 3:** Summary of TiO_2_ nanotubes based materials in photoelectrocatalytic water splitting

Photocatalyst	Light intensity	Electrolyte	Photocurrent (mA cm^–2^)	IPCE and water splitting rate	Ref.
TiO_2_ NTAs	150 mW cm^–2^ Xe lamp	1 M KOH + 0.5 M H_2_SO_4_	7.0	η = 4.39% at –5.4 V_SCE_	86
				2.53 mL cm^–2^ h^–1^	
TiO_2_ NTAs	110 mW cm^–2^ 350 W Xe lamp	1 M KOH + 0.5 M H_2_SO_4_	4.95 at 0 V_SCE_	η = 4.13% at –0.64 V_SCE_	248
				97 μmol cm^–2^ h^–1^	
TiO_2_ NTAs with two‐step anodization	10 mW cm^–2^ 500 W Xe lamp	10 v% KOH + 90 v% EG	0.67 at 0.5 V_RHE_	η = 9.75%	292
				0.08 mL cm^–2^ h^–1^ at 0.5 V_RHE_	
TiO_2_ NTAs with three‐step anodization	300 W Xe lamp	2 M Na_2_CO_3_ + 0.5 M EG	24	420 μmol cm^–2^ h^–1^ at –0.3 V_SCE_	265
TiO_2_ NTAs	110 mW cm^–2^ 350 W Xe lamp	1 M KOH + 0.5 M H_2_SO_4_	5.8 at 0 V_SCE_	η = 4.49% at –0.46 V_SCE_	266
				122 μmol cm^–2^ h^–1^	
TiO_2_ NTAs	100 mW cm^–2^ 300 W Xe lamp	1 M KOH	1.55 at 0.6 V_Ag/AgCl_	η = 1.13%	267
				0.57 mL cm^–2^ h^–1^ at 0.6 V_Ag/AgCl_	
TiO_2_ NTAs fabricated in HCl electrolytes	100 mW cm^–2^ AM 1.5	HCl + H_2_O_2_ + EG	0.65	η = 0.42% 391 μL cm^–2^ h^–1^	269
Black TiO_2_ NTAs	100 mW cm^–2^ AM 1.5	1 M NaOH	3.65 at 0.23 V_Ag/AgCl_	IPCE = 80% at 360 nm	273
				η = 1.20% at 0.23 V_Ag/AgCl_	
SrTiO_3_/TiO_2_ NPs/TiO_2_ NTAs	320 mW cm^–2^ 300 W Xe lamp	0.5 M KOH and 0.5 M EG	1.91 at 0.3 V_SCE_	IPCE = 100% at 325 nm	58
				314.9 μmol cm^–2^ h^–1^ at 0.3 V_SCE_	
Pd/TiO_2_ NTAs	320 mW cm^–2^ 300 W Xe lamp	2 M Na_2_CO_3_ + 0.5 M EG	26.8 at 0.9 V_SCE_	IPCE = 100% at 330 nm	135
				592 μmol cm^–2^ h^–1^ at 0.9 V_SCE_	
Pt/TiO_2_ NTAs	30 mW cm^–2^ (λ > 400 nm)	0.1 M Na_2_SO_4_ + 1 M EG	0.046 at 0 V_Ag/AgCl_	120 μmol cm^–2^ h^–1^ at 0 V_Ag/AgCl_	188
Carbon QDs/TiO_2_ NTAs	100 mW cm^–2^ AM 1.5	0.25 M Na_2_S + 0.35 M Na_2_SO_3_	1.0 at 0 V_Ag/AgCl_	IPCE = 28% at 410 nm	205
				14.1 μmol cm^–2^ h^–1^ at 0 V_Ag/AgCl_	
CdSe/CdS/TiO_2_ NTAs	100 mW cm^–2^ AM 1.5	10 v% EG + 90 v% Na_2_S	10 at 0.5 V_SCE_	η = 9.47%	257
				10.24 mL cm^–2^ h^–1^ at 0.5 V_SCE_	
Au/RGO/TiO_2_ NTAs	Visible light (λ > 400 nm)	1 M KOH	0.224 at 1.23 V_RHE_	IPCE = 5.8% at 580 nm	275
				45.0 μmol cm^–2^ h^–1^ at 1.23 V_RHE_	
CdS/TiO_2_ NTAs	300 W Xe lamp (λ > 400 nm)	0.2 M Na_2_S	0.25 at 0.5 V_SCE_	0.34 ml cm^–2^ h^–1^ at 0.5 V_SCE_	276
TiO_2_ NTAs	113 W cm^–2^	10 v% KOH+ 90 v% EG	6.6 at 0 V_SCE_	4.4 mL cm^–2^ h^–1^ at 0.5 V_SCE_	289
TiO_2_ NTAs	35 mW cm^–2^ (350< λ <450 nm)	0.5 M H_2_SO_4_	8.1 mA	IPCE = 25% at 344 nm	290
				0.152 mmol h^–1^	
TiO_2_ NTAs	150 mW cm^–2^ 350 W Xe lamp	1 M KOH + 0.5 M H_2_SO_4_	5.5	η = 3.51% at –0.6 V_SCE_	291
				93.6 μmol cm^–2^ h^–1^ at –0.61 V_SCE_	
Pd QDs/TiO_2_ NTAs	35 mW cm^–2^ solar light	0.5 M KOH + 0.1 M glucose	1.1 at –0.3 V_SCE_	27.5 μmol cm^–2^ h^–1^ at –0.3 V_SCE_	293
Fe/TiO_2_ NTAs	100 mW cm^–2^ Xe lamp	1 M NaOH	1.32 at 1.5 V_Ag/AgCl_	10 μL cm^–2^ h^–1^ at 1.5 V_Ag/AgCl_	294
Au/TiO_2_ NTAs	Visible light (λ > 400 nm)	0.1 M EDTA‐2Na +0.2 M Na_2_SO_4_	1.7	4.5 μmol cm^–2^ h^–1^	295
S/TiO_2_ NTAs	100 mW cm^–2^ 150 W Xe lamp	0.1 M KOH	2.92 at 1.0 V_Ag/AgCl_	IPCE = 43% at 350 nm	296
				IPCE = 2.4% at 500 nm at 1.0 V_Ag/AgCl_	
Polypyrrole/TiO_2_ NTAs	100 mW cm^–2^ AM 1.5	1 M KOH	3.48 at 0.23 V_Ag/AgCl_	IPCE = 92.5% at 300 nm	297
				28.8 μmol cm^–2^ h^–1^ at 0.23 V_Ag/AgCl_	
ZnO/TiO_2_ NTAs	100 mW cm^–2^ Xe lamp	1 M NaOH	1.24 at 1.5 V_Ag/AgCl_	11 μL cm^–2^ h^–1^ at 1.5 V_Ag/AgCl_	298
C/N/TiO_2_ NTAs	3.12 mW cm^–2^ Hg lamp	H_2_O	3.0 at 1.0 V_SCE_	11 mmol cm^–2^ h^–1^ at 1.0 V_SCE_	299
Au/TiO_2_ NTAs	150 W Xe lamp	0.5 M Na_2_SO_4_	0.039 at 0.5 V_Ag/AgCl_	η = 0.045% at 0.1 V_SCE_	300
WO_3_/TiO_2_ NTAs	100 mW cm^–2^ 200 W Xe lamp	1 M KOH	0.62 at 0.2 V_Ag/AgCl_	1.07 mL cm^–2^ h^–1^ at 0.2 V_Ag/AgCl_	301
N/Ni/TiO_2_ NTAs	100 mW cm^–2^ 150 W Xe lamp	1 M KOH	2.52 at 0 V_Ag/AgCl_	η = 1.12% at –0.52 V_Ag/AgCl_	302
Ti^3+^/TiO_2_ NTAs	100 mW cm^–2^ AM 1.5	1 M KOH	3.05 at 1.23 V_RHE_	η = 1.66% at 0.78 V_RHE_	303
				IPCE = 82.8% at 350 nm	
RGO/Ti^3+^/TiO_2_ NTAs	100 mW cm^–2^ 500 W Xe lamp	1 M KOH	1.44 at 1.23 V_RHE_	IPCE = 96.2% at 350 nm at 1.23 V_RHE_	304
Al_2_O_3_/TiO_2_ NTAs	300 mW cm^–2^ AM 1.5	1 M KOH	0.9 at 0.5 V_Ag/AgCl_	IPCE = 70% at 360 nm at 0.5 V_Ag/AgCl_	305
N/C/H–TiO_2_ NTAs	100 mW cm^–2^ AM 1.5	1 M KOH	3.6 at 0.23 V_Ag/AgCl_	IPCE = 64.5% at 330 nm at 0.23 V_Ag/AgCl_	306
CdSe/TiO_2_ NTAs	Visible light (λ > 400 nm)	1 M NaOH	0.14 at 0 V_Ag/AgCl_	IPCE = 0.45% at 550 nm at 0 V_Ag/AgCl_	307
Ag_2_S/TiO_2_ NTAs	25 mW cm^–2^ (λ > 385 nm)	0.5 M Na_2_S	0.84 at 0 V_Ag/AgCl_	IPCE = 20% at 600 nm at 0 V_Ag/AgCl_	308
WO_3_/TiO_2_ NTAs	100 mW cm^–2^	0.1 M H_2_SO_4_+1 MeOH	3.5 at 1.6 V_Ag/AgCl_	IPCE = 50% at 370 nm at 1.6 V_Ag/AgCl_	309
Hydrogenated TiO_2_ nanotubes	100 mW cm^–2^ AM 1.5	1 M NaOH containing 1 wt% of EG	0.65 at 0 V_Ag/AgCl_	η = 0.30%	310
				IPCE = 74% at 370 nm at 0 V_Ag/AgCl_	
W/TiO_2_ NTAs	75 mW cm^–2^ 300 W Xe lamp	0.1 M Na_2_S + 0.02 M Na_2_SO_3_	3.06 at 1.0 V_SCE_	η = 7.3%	311
				24.97 μmol cm^–2^ h^–1^ at 1.0 V_SCE_	
WO_3_/TiO_2_ NTAs	800 W m^–2^ 150 W Xe lamp	1 M KOH containing 1 wt% of EG	2.4 at 0.6 V_SCE_	22 mL cm^–2^ h^–1^ at 0.6 V_SCE_	312
Hierarchical TiO_2_ NTAs	87 mW cm^–2^ 300 W Xe lamp	1 M KOH +10 v% EG	1.78 at 1.23 V_Ag/AgCl_	η = 1.84%	313
				0.87 mL cm^–2^ h^–1^ at 1.23 V_Ag/AgCl_	
C/K/TiO_2_ NTAs	Visible light (λ > 400 nm)	1 M KOH	5.0 at 0.3 V_SCE_	η = 2.5%	314
				10.98 μL cm^–2^ h^–1^ at 0.3 V_SCE_	
Reduced TiO_2_ NTAs	370 mW cm^–2^ (λ > 400 nm)	1 M NaOH	0.732 at 1.23 V_RHE_	η = 1.31% at 0.4 V_RHE_	315
				IPCE = 68.7% at 330 nm	
				13.75 μmol h^–1^ at 1.23 V_RHE_	
Au/TiO_2_ NTAs	Visible light (λ > 400 nm)	1 M KOH	150 at 1.23 V_RHE_	IPCE = 7.9% at 556 nm	316
				η = 1.1%	
				27.9 μmol h^–1^ at 1.23 V_RHE_	
Pt/TiO_2_ NTAs	320 mW cm^–2^ 300 W Xe lamp	2 M Na_2_CO_3_ + 0.5 M EG	24.2 at –0.3 V_SCE_	IPCE = 87.9% at 350 nm	317
				592 μmol cm^–2^ h^–1^ at –0.3 V_SCE_	
CdS/TiO_2_ NTAs	100 mW cm^–2^ AM 1.5	1 M Na_2_S	4.8	η = 2.58%	318
				1.12 mL cm^–2^ h^–1^	

## 6 Conclusions

The fast depletion of fossil fuel and serious environmental problems have been hot topics in recent years. Solar water splitting accompanied with photocatalytic pollutant degradation is recognized as a potential science and technology advancement for solving these issues in the future. Until now, a large number of fundamental studies on synthesis, modification and applications have been extensively carried out. Great progress has been made for 1D TiO_2_ nanostructured materials at the laboratory scale. In this review, we present the state‐of‐the‐art development on the fabrication 1D TiO_2_ nanotubes with a well‐controlled size and morphology by hydrothermal method, solvothermal method, template technique and electrochemical anodic oxidation. Among them, the hydrothermal and anodization route are most popular for the synthesis of 1D TiO_2_ nanotubes due to the easy operation and rational control of the nanostructures. Besides, it is necessary to construct heterostructures by modifying or doping with metal, non‐metal and semiconductors in order to achieve sufficient efficiency. At last, this review discussed the factors such as sacrificial agent (methanol, ethanol, KOH etc.), light intensity, the applied potential, TiO_2_ morphology and structure which have an influence on the photo/photoelectron‐catalytic hydrogen production rate. Owing to low‐cost production, high aspect ratio, large specific surface area, and excellent electronic or ionic charge transfer properties, 1D TiO_2_ nanotubes are widely used in photocatalytic degradation of pollutants, water splitting, solar cells, photoreduction of CO_2_, supercapacitors and lithium‐ion batteries.

However, there are still extensive scientific and technical challenges on the functionalities and performances of 1D TiO_2_ nanotubes in the field of solar water splitting. As a wide band gap material, TiO_2_ showed a low utilization of solar light and fast recombination of electro/hole pairs, resulting in low solar‐to‐hydrogen efficiency and photocatalytic hydrogen generation activity. Therefore, more efforts should be focused on investigating the synergistic effects of the different engineering strategies including increasing the specific surface area, modifying with other novel low‐cost nanomaterials with improved light harvest and photoelectric conversion performances. Meanwhile, it is meaningful and promising to prepare TiO_2_ composites to realize photocatalytic water splitting for hydrogen production and photocatalytic degradation of pollutants at the same time. Since titanate materials possess large interlayer spacing and adjustable lattice parameters, it is essential to obtain the titania materials with unique ion‐exchange properties and required crystal structures by rational control of the morphology of the starting material. Moreover, by combining the excellent adsorption ability of titanates and high photocatalytic activity of titania, faster photocatalytic water splitting or photodegradation of organic pollutants in wastewater can be significantly achieved. Besides, the introduction of biologically active materials to TiO_2_ is also a good choice to explore new materials and versatile methods for developing high performance solar‐to‐fuel devices with sustainable properties. Additionally, most of the researches are still in the laboratory and the output of H_2_ falls far from the needs of industrial production. So scaling up of the photocatalytic/photoelectrochemical instrument/equipment should be developed to make these technologies applicable for practical application as soon as possible.

Morever, there is a long way to go to produce hydrogen without sacrificial agents in water. Equally challenging is to decrease the reduction overpotential needed for water splitting. With all the challenges of the TiO_2_ photocatalyst, we expect dimensionally constructed TiO_2_ materials be able to play an important role in the future clean fuel hydrogen generation and environmental remediation applications. Thus, with further development of new technology and more efficient semiconductor materials, commercialization of 1D TiO_2_‐based materials for efficient hydrogen production is achiverable in the near future.
